# A toolkit for facilitating markerless integration of expression cassettes in *Komagataella phaffii* via CRISPR/Cas9

**DOI:** 10.1186/s12934-025-02716-x

**Published:** 2025-05-03

**Authors:** Laura García-Calvo, Charlotte Kummen, Solvor Rustad, Sissel Beate Rønning, Annette Fagerlund

**Affiliations:** Nofima – Norwegian Institute of Food, Fisheries and Aquaculture Research, Ås, Norway

**Keywords:** *Pichia pastoris*, *Komagataella phaffii*, CRISPR/Cas9, Heterologous expression, Golden Gate assembly, Food proteins, Ovalbumin, WGS

## Abstract

**Background:**

The yeast *Komagataella phaffii* (formerly known as *Pichia pastoris*) has been widely used for functional expression of recombinant proteins, including plant and animal food proteins. CRISPR/Cas9 genome editing systems can be used for insertion of heterologous genes without the use of selection markers. The study aimed to create a convenient markerless knock-in method for integrating expression cassettes into the chromosome of *K. phaffii* using CRISPR/Cas9 technology. The approach was based on the hierarchical, modular, Golden Gate assembly employing the Golden*Pi*CS toolkit. Furthermore, the aim was to evaluate the system’s efficiency and suitability for producing secreted recombinant food proteins.

**Results:**

Three Cas9/sgRNA plasmids were constructed, along with corresponding donor helper plasmids containing homology regions for chromosomal integration via homology-directed repair. The integration efficiency of an enhanced green fluorescent protein (eGFP) expression cassette was assessed at three genomic loci (*04576*, *PFK1*, and *ROX1*). The *04576* locus showed the highest integration efficiency, while *ROX1* had the highest transformation efficiency. Whole genome sequencing revealed variable copy numbers of *eGFP* expression cassettes among clones, corresponding with increasing levels of fluorescence. Furthermore, the system’s applicability for producing recombinant food proteins was validated by successfully expressing and secreting chicken ovalbumin. This constitutes the first report of CRISPR/Cas9 applied to produce recombinant chicken ovalbumin.

**Conclusions:**

The adapted Golden*Pi*CS toolkit combined with CRISPR/Cas9 technology enabled efficient and precise genome integration in *K. phaffii*. This approach holds promise for expanding the production of high-value recombinant proteins. Future research should focus on optimizing integration sites and improving cloning procedures to enhance the system’s efficiency and versatility.

**Supplementary Information:**

The online version contains supplementary material available at 10.1186/s12934-025-02716-x.

## Background

*Komagataella phaffii* (formerly known as *Pichia pastoris*) is a methylotrophic yeast that has been widely used for functional expression of heterologous genes [1]. Several animal and plant food proteins produced recombinantly in this expression host have been approved for sale in the U.S. market in recent years, through their classification as ‘generally recognized as safe’ (GRAS) [2–5]. Production of recombinant food proteins using microorganisms as cell factories, termed precision fermentation, has been suggested as a promising source of novel protein ingredients in future food systems [6–9].

Benefits of using *K. phaffii* as an expression host include a high secretion capacity and low level of endogenous secreted proteins, facilitating purification of heterologous proteins from the culture medium. Other advantages include its ability to perform many post-translational modifications typically associated with higher eukaryotes, as well as the availability of a comprehensive collection of molecular biology tools and toolkits for genetic manipulation [1, 10–13], including a wide range of characterised promoters [14, 15].

Established classical expression systems in *K. phaffii* usually employ integrative vectors incorporated into the genome by single-crossover homologous recombination. Due to their instability in this host, there has been limited use of episomal plasmids. Both approaches, however, require selection markers, which may be a disadvantage. For example, the use of antibiotic resistance markers is incompatible with production of food proteins, and auxotrophic strains show growth impediments [16, 17]. Furthermore, the use of integrative vectors generates high clonal variability, due to, among others, variations in the copy number of the inserted vectors and a tendency for nonhomologous integration at random chromosomal locations. This necessitates labour-intensive screening for identification of suitable expression clones [18, 19].

More recently, CRISPR/Cas9 (clustered regularly interspaced short palindromic repeats/CRISPR associated protein 9) systems have been established and optimised for use in *K. phaffii* [20–22]. The systems require cloning of a single-guide RNA (sgRNA), which guides Cas9 to the target sequence where it introduces a double-strand break, into an sgRNA expression cassette on an episomal plasmid, from which human codon-optimised Cas9 is also expressed. In the CRISPR/Cas9 system described by Gassler et al. [22], the sgRNA is flanked by 5′ self-splicing hammerhead and 3′ hepatitis delta virus ribozyme sequences for correct processing [23]. By providing a homologous repair template containing the sequences upstream and downstream of the sgRNA-guided Cas9 cleavage site (homology regions), precise genome modifications can be obtained, including the insertion of heterologous genes by double crossover cassette exchange. The precise nature of the insertions makes screening of a large number of expression clones unnecessary. The process can be very efficient, precluding the need for selection markers on the integration cassettes [21].

A major challenge for the effective application of the CRISPR/Cas9 system is the identification of target sites that can be efficiently cleaved, despite the existence of multiple computational tools to assist in the design of sgRNAs [24]. Large differences in targeting efficiency have been demonstrated when testing different sgRNAs for CRISPR/Cas9 systems, also in *K. phaffii* [11, 20, 21]. It was for example shown that the efficiency of gene disruptions obtained with two different sgRNAs targeting the same gene, using the CRISPR/Cas9 system employed in the current study, varied from 0 to 100% [11]. Another challenge for heterologous gene expression is the identification of neutral integration sites which allow robust expression of the exogenous gene(s), without affecting cell fitness. Only a few sites for integration are routinely used in *K. phaffii*. Brady et al. [25] developed a framework for identification of suitable integration sites using ATAC-seq, which was demonstrated in *K. phaffii*. By assaying the expression levels of an enhanced green fluorescent protein (eGFP) reporter, they showed that the performance of integration sites varied, and identified neutral sites mediating high efficiency expression of exogenous genes. The same group also showed that genes could successfully be targeted to intragenic regions of the genome adjacent to genes *GQ67_04576*, *PFK1*, and *ROX1*, using 500 bp flanking sequences for homology-directed repair [21]. Other studies have also screened intergenic regions and identified neutral genomic integration sites displaying varying integration and expression efficiencies [26–28].

Generation of efficient heterologous expression strains usually involves combinatorial expression optimization including screening of different promoters and genome integration sites in order to achieve the best possible output. Prielhofer et al. [11] developed a convenient modular toolkit based on Golden Gate cloning, named Golden*Pi*CS (Golden Gate derived *P. pastoris* cloning system), for the assembly of expression cassettes on integrative plasmids. It is based on previously developed modular cloning systems MoClo [29] and GoldenMOCS [30]. Golden Gate cloning employs type IIs restriction enzymes (e.g., *Bsa*I and *Bpi*I) which cut outside of their recognition site, giving four base pair overhangs that can be freely designed, enabling scarless cloning of multiple inserts in a single reaction [31]. The overhangs are referred to as ‘fusion sites’. The Golden*Pi*CS toolkit was designed to enable assembly of promoter, target gene, and terminator modules into transcription units on an integrative plasmid [11]. Three hierarchical backbone (BB) levels are used for assembly of transcription units prior to integration. Each level employs defined fusion sites. In the first level (BB1), the basic modules to be assembled—promoters, target gene, and terminator—are each cloned into BB1 plasmids using *Bsa*I Golden Gate assembly reactions. In the second level (BB2), the three genetic elements are assembled into a single transcription unit by *Bpi*I cloning into a BB2 recipient plasmid. Multiple transcription units are then assembled into multigene constructs by *Bsa*I cloning into a third level backbone plasmid (BB3), used for genomic integration. If only one expression unit is to be inserted, the BB2 level is bypassed, and the unit is instead inserted into a BB3 plasmid designed for *Bpi*I cloning. In the Golden*Pi*CS toolkit, the BB3 level plasmid is an integrative plasmid, with the choice of four antibiotic selection markers and three integration loci for single crossover genomic integration into *K. phaffii* (*AOX1tt*, *ENO1*, and *RG12*).

The current study aimed to adapt the Golden*Pi*CS toolkit [11] for use with CRISPR/Cas9, expanding its use to convenient cloning of one or more expression units to be inserted into neutral genomic sites shown to be successfully targeted using CRISPR/Cas9 technology. The three selected integration sites were those used by Dalvie et al. [21], namely *GQ67_04576* (hereafter referred to as *04576*), *PFK1*, and *ROX1*. The approach leveraged the Golden Gate-based modular cloning system provided in Golden*Pi*CS to facilitate convenient generation of donor cassettes used for chromosomal integration via homology-directed repair. Donor helper plasmids were designed to assemble the donor expression cassettes, and included homology regions for double crossover cassette exchange. These helper plasmids replaced the BB3 level integrative plasmids with antibiotic resistance markers from Golden*Pi*CS. Thus, three pairs of plasmids were constructed, each comprising a Cas9/sgRNA plasmid and a corresponding donor helper plasmid. The system’s targeting efficiency and relative protein expression levels were evaluated by integrating an *eGFP* expression cassette and performing whole genome sequencing (WGS) analysis. Additionally, the generation of an ovalbumin (egg protein) expression strain demonstrated the system’s suitability for producing secreted recombinant food proteins.

## Results

### Design of sgRNA plasmids and crBB3 donor helper plasmids

**Fig. 1:**
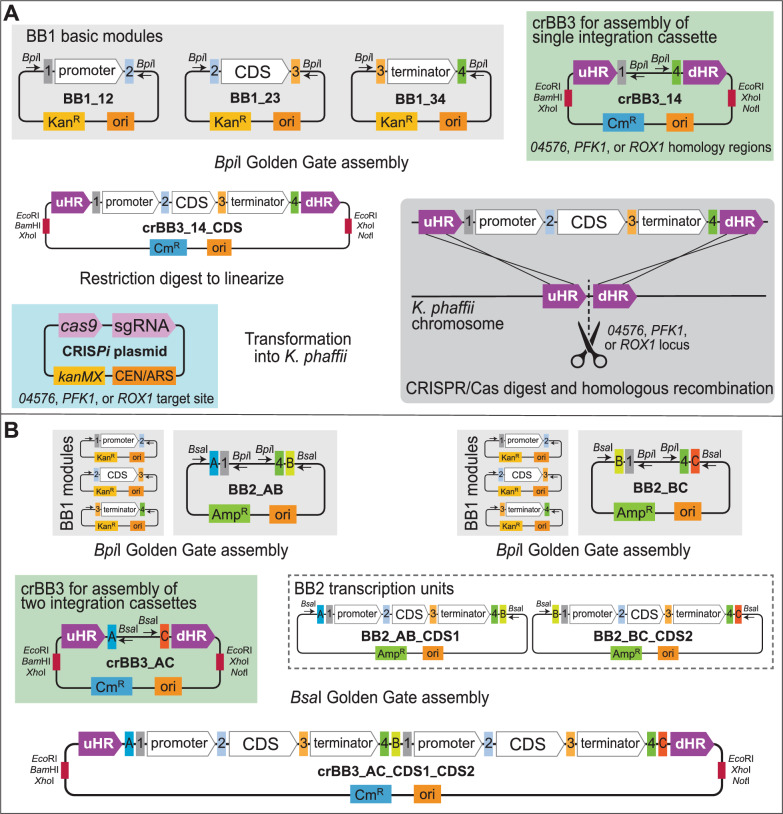
Plasmids and cloning strategy. The backbone 1 (BB1) modules from the Goldern*Pi*CS kit were used. Additional modules can be inserted into empty BB1 plasmids using *Bsa*I Golden Gate Assembly (GGA) and fusion sites Fs1 (GGAG), Fs2 (CATG), Fs3 (GCTT), Fs4 (CGCT). **A** The genetic elements on BB1 plasmids were combined into a single transcription unit by *Bpi*I GGA into a recipient crBB3_14 donor helper plasmid, containing homology regions for *04576*, *PFK1*, or *ROX1* target sites (uHR and dHR). The resulting plasmid was linearized and co-transformed with the corresponding CRIS*Pi* plasmid harbouring Cas9 and the sgRNA targeting the appropriate locus into *K. phaffii* host cells. **B** Multigene constructs can be prepared by assembling single transcription units into empty BB2 level plasmids using *Bpi*I GGA. Fusion sites FsA (GATC), FsB (CCGG), FsC (AATT), etc. are then used to insert the expression cassettes into BB3 plasmids (e.g., crBB3_AC) using *Bsa*I GGA

The CRISPR/Cas9 system described by Gassler et al. [22] was used to generate plasmids co-expressing an sgRNA and the human codon optimised Cas9 enzyme, to target double-strand breaks at each of the three integration sites *04576*, *PFK1*, and *ROX1*. These three integration sites correspond to intragenic regions of the genome selected based on a previous study [21], where they were shown to be neutral integration sites that could be successfully targeted with CRISPR/Cas9 technology. The plasmids were named CRIS*Pi*_04576, CRIS*Pi*_PFK1, and CRIS*Pi_*ROX1, respectively (Table 1 and Fig. 1A). Transient episomal plasmid maintenance in *K. phaffii* was mediated by the *Saccharomyces cerevisiae* CEN6/ARS4 locus and a geneticin resistance marker.

**Table 1:** Plasmids constructed in the current study

Plasmid name	Clone no	Description	Selection marker
CRIS*Pi*_04576	MF8295	Shuttle vector for sgRNA/Cas9 targeting near *04576*	*kanMX*
CRIS*Pi*_PFK1	MF8296	Shuttle vector for sgRNA/Cas9 targeting near *PFK1*	*kanMX*
CRIS*Pi*_ROX1	MF8297	Shuttle vector for sgRNA/Cas9 targeting near *ROX1*	*kanMX*
crBB3_14_04576	MF8310	Helper plasmid for cloning of one transcription unit with *04576* homology regions	*cat*
crBB3_14_PFK1	MF8311	Helper plasmid for cloning of one transcription unit with *PFK1* homology regions	*cat*
crBB3_14_ROX1	MF8312	Helper plasmid for cloning of one transcription unit with *ROX1* homology regions	*cat*
crBB3_AC_04576	MF8437	Helper plasmid for cloning of two transcription units with *04576* homology regions	*cat*
crBB3_AC_PFK1	MF8438	Helper plasmid for cloning of two transcription units with *PFK1* homology regions	*cat*
crBB3_AC_ROX1	MF8439	Helper plasmid for cloning of two transcription units with *ROX1* homology regions	*cat*
crBB3_04576_eGFP	MF8316	crBB3_14_04576 with inserted *eGFP* expression cassette	*cat*
crBB3_PFK1_eGFP	MF8317	crBB3_14_PFK1 with inserted *eGFP* expression cassette	*cat*
crBB3_ROX1_eGFP	MF8318	crBB3_14_ROX1 with inserted *eGFP* expression cassette	*cat*
pEX-A128-αMF	MF8448	Synthesized α-MF, template for PCR	*bla*
pTOPO-αMF	MF8970	α-MF for fusion to *OVA*, cloned in pCR4Blunt-TOPO	*bla*, *kanMX*
pEX-K248-OVA		Synthesized *OVA* gene	*kanMX*
crBB3_04576_OVA	MF8982	crBB3_14_04576 with inserted *OVA* expression cassette	*cat*

Backbone (BB) donor helper plasmids corresponding to the BB3 level in the hierarchical cloning system employed in the Golden*Pi*CS toolkit [11] (crBB3 plasmids) were generated for cloning of one or two expression units (Table 1). The donor plasmids harboured either *Bpi*I restriction sites and fusion sites Fs1 and Fs4 for cloning of one transcription unit (crBB3_14 plasmids; Fig. 1A), or *Bsa*I restriction sites and fusion sites FsA and FsC for cloning of two transcription units (crBB3_AC plasmids, Fig. 1B). The cloning sites were flanked by 500 bp sequences for homology-directed repair (homology regions) comprising the sequences upstream and downstream of each CRISPR/Cas9 targeting site. To insert three or more transcription units, the existing linkers may be replaced with linkers containing fusion sites FsA-FsD, FsA-FsE, and so on. A hairpin was inserted upstream of the cloning sites, to minimize any transcriptional interference from upstream endogenous genes following insertion into the *K. phaffii* genome. The donor plasmids may be linearized prior to transformation into *K. phaffii* using *Eco*RI, *Xho*I, *Bam*HI, or *Not*I restriction digestion.

The crBB3 plasmids can be used for *Bpi*I or *Bsa*I Golden Gate assembly of any sets of inserts between the homology regions, providing that the outermost fragments harbour the Fs1/Fs4 (GGAG/CGCT) or FsA/FsC (GATC/AATT) fusion sites, respectively. All fragments to be assembled should be devoid of the type IIs restriction enzyme recognition sites (*Bpi*I and/or *Bsa*I), as well as at least one of the restriction sites used for linearization of the donor plasmid prior to transformation.

### Transformation and integration efficiencies differed between sites

To evaluate the efficiency of integration using the three chosen target sites, three donor DNA cassettes for expression of *eGFP* were constructed. The selected promoter and terminator modules were both from the *K. phaffii THD3* gene. The *eGFP* expression cassette was inserted into the three crBB3_14 donor helper plasmids containing homology regions for integration near genes *04576*, *PFK1*, and *ROX1*, generating donor cassette plasmids crBB3_04576_eGFP, crBB3_PFK1_eGFP, and crBB3_ROX1_eGFP, respectively (Table 1).

**Fig. 2:**
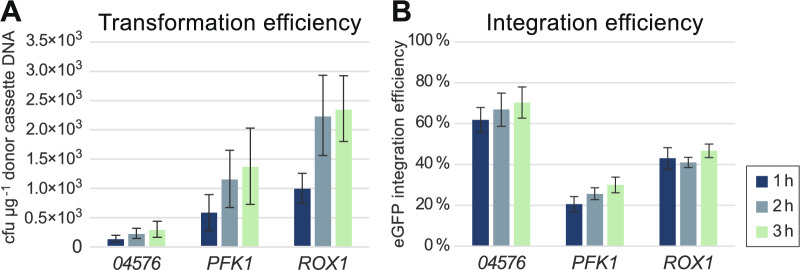
Efficiency of CRISPR/Cas9-mediated markerless eGFP donor cassette integrations targeting *04576*, *PFK1*, and *ROX1* loci. **A** Transformation efficiency shown as the total number of transformants (cfu µg^−1^ donor cassette DNA). **B** Integration efficiency shown as the percentage of transformants with integrated eGFP, determined by measuring green fluorescence for single clones in an endpoint screening assay after 48 h of growth in 96-well plates. Transformation reactions were incubated for 1, 2, and 3 h prior to selection on G418 agar. Data shown are mean values for three independent experiments with, in total, n = 2234 tested clones, and error bars show standard errors of the means

*K. phaffii* was co-transformed with donor cassette plasmids linearized by *Eco*RI digestion (2 µg, corresponding to 1.1 µg donor cassette DNA) and CRIS*Pi* plasmids containing the appropriate sgRNA (100 ng). Transformation reactions were selected for clones carrying CRIS*Pi* plasmid by plating on selective agar after 1, 2, and 3 h of recovery after electroporation. Integration of *eGFP* expression cassettes was evaluated by screening of clones for green fluorescence in an endpoint assay after 48 h of growth (Fig. 2). A total of 2234 clones were tested, up to 100 clones for each reaction and time-point.

The transformation efficiencies differed significantly between integration sites (Tukey’s post hoc test, *p* ≤ 0.015), with the lowest number of transformants obtained at locus *04576* (Fig. 2A). However, reactions targeting the *04576* site obtained the highest integration efficiencies, with an average of 66% green fluorescent clones (Fig. 2B). Integration efficiencies also differed significantly across integration sites (Tukey’s post hoc test, *p* < 0.001).

Integration of *eGFP* into the *04576* locus resulted in 150 (SE ± 49) successful integrations per µg donor cassette DNA, while the corresponding results for the *PFK1* and *ROX1* loci were 289 (SE ± 88) and 812 (SE ± 158), respectively (average values across all time-points). Both transformation and integration efficiencies generally increased slightly from 1 to 3 h of recovery after electroporation (Fig. 2), although the increase in integration efficiency was not statistically significant (ANOVA, *p* = 0.086). However, there was a significant difference in transformation efficiency between the 1 and 3 h time-points (Tukey’s post hoc test, *p* < 0.022).

### Expression of secreted ovalbumin

The potential of the presented method for generating recombinant microbial strains for food applications was evaluated through the extracellular production of chicken egg ovalbumin, an ingredient of high relevance for the food industry due to its nutritional and functional properties [32]. Golden*Pi*CS was designed mainly for the engineering of metabolic pathways [11], rather than for expressing heterologous proteins for secretion. Consequently, the kit does not contain BB1-level modules for secretion signal sequences. For secreted proteins, the secretion signal and the gene encoding the mature protein may be combined in a Golden Gate assembly reaction into the empty backbone plasmid BB1_23, or synthesized as a complete gene cassette containing both a compatible signal sequence and the mature coding sequence. Alternatively, two separate BB1-level plasmid modules can be designed for the secretion signal and the coding sequence, which can then be simultaneously inserted into the BB2 or BB3-level vector backbones. This latter approach, which facilitates testing different signal sequences (and can be expanded to include a signal peptide library [12]), was used in the current study to construct an expression cassette for the chicken ovalbumin gene (*OVA*) (Supplementary Figure S1). The *S. cerevisiae* α-mating factor (α-MF) sequence, carrying the L42S mutation mediating increased protein secretion [33], was selected as the secretion signal. The selected promoter and terminator were the same as for the *eGFP* expression cassette, and the selected target locus was *04576*.

Co-transformation into *K. phaffii* with the CRIS*Pi*_04576 plasmid (100 ng) and the linearized donor cassette plasmid (1.65 µg, corresponding to 1 µg donor cassette DNA) resulted in 143 and 157 colony forming units (cfu) per µg donor cassette DNA after 2 and 3 h of recovery, respectively. This transformation efficiency was slightly lower than that obtained for insertion of *eGFP* in the *04576* integration site, in which on average 214 and 283 cfu µg^−1^ donor cassette DNA were obtained at the corresponding time-points. PCR screening of randomly selected *OVA* expression cassette transformants (n = 61) showed that the overall targeting efficiency was 47%, increasing from 40 to 51% after 2 and 3 h of recovery.

**Fig. 3:**
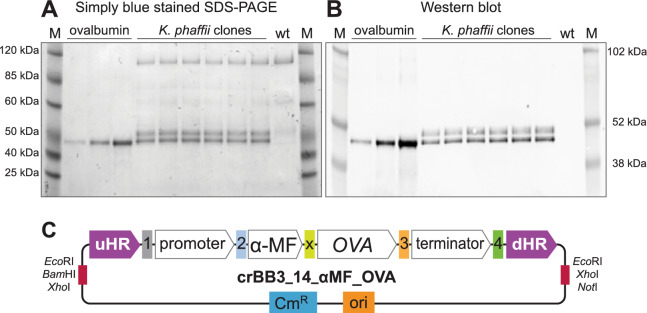
Recombinant ovalbumin secreted into the culture supernatant. Representative gels and blots are shown. **A** SDS-PAGE gel. **B** Anti-ovalbumin Western blot. The reference is hen egg ovalbumin at concentrations 12.5, 25, and 50 ng µL^−1^ and wt contains wild type *K. phaffii* ATCC 76273 supernatant. Thirteen µL supernatant or reference protein was applied to each well. M: Molecular weight markers. **C** crBB3 donor helper plasmid construct after Golden Gate assembly of the modules constituting the *OVA* expression cassette. The fusion site labelled X linking the α-MF sequence with the downstream codon-optimised *OVA* sequence in the *Bpi*I cloning reaction constitutes the last four bases of the α-MF coding sequence (AGCT)

Twelve clones were examined for recombinant ovalbumin protein production by growth in 50 mL defined medium for 48 h in shake flasks, to optical density at 600 nm (OD600) of 25.5 (SE ± 0.38). SDS-PAGE and Western blots for supernatant samples showed that two proteins detected by the anti-OVA antibodies, of approximate sizes 45 and 47 kDa, were secreted into the culture medium in all clones (Fig. 3). Based on previous studies [34, 35], the two bands are expected to represent ovalbumin variants with different glycosylation patterns. The mean calculated titer (including both bands) was 35 (SE ± 1.3; range 26–40) mg L^−1^ and the OVA titer normalized to biomass was 1.38 (SE ± 0.06; range 1.1–1.7) mg L^−1^ OD600^−1^. This shows that there was limited variation in protein expression levels between the 12 examined clones, and constitutes a proof of concept that the current approach may be used to produce secreted food proteins.

### Fluorescence intensity differed between *eGFP* expression clones

**Fig. 4:**
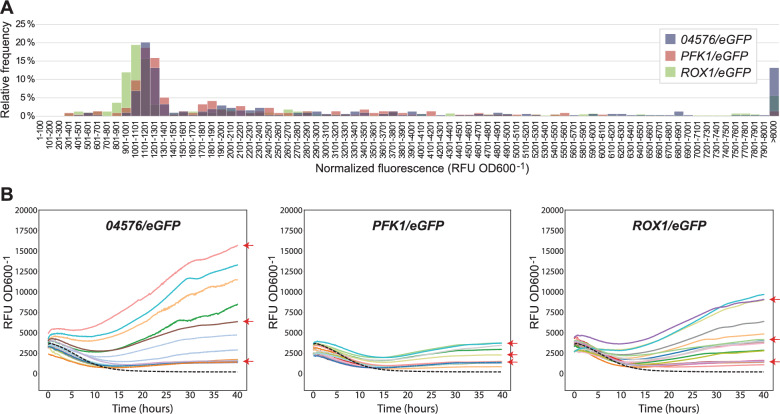
Differences in fluorescence levels for clones with integrated eGFP, for each of the three target sites. **A** Distribution of fluorescence levels determined by an endpoint screening assay after 48 h of growth. Data is shown as overlayed relative frequency histograms. All fluorescent clones with OD600 > 1 (n = 892) were included in the analysis. **B** Kinetic fluorescence growth assay for selected fluorescent clones (19 to 23 clones for each target site). *Arrows* indicate the nine isolates with different fluorescence levels (low, medium, strong; respectively, *left panel*: *04576*_35, *04576*_38, *04576*_37, *centre panel*: *PFK1*_72, *PFK1*_66, *PFK1*_21, *right panel*: *ROX1*_96, *ROX1*_87, *ROX1*_97) selected for WGS. Each growth curve shows averages of 2 to 4 biological replicates. A plot displaying standard deviations for each curve is shown in Supplementary Figure S3. Normalized fluorescence (RFU OD600^−1^) is defined as relative fluorescence units (RFU) relative to culture optical density at 600 nm (OD600). The discontinuous *black line* corresponds to the wild-type control

Next, it was of interest to examine whether protein expression levels varied between the *04576*, *PFK1*, and *ROX1* target sites, and/or among different clones for the same integration site. For this analysis, the data from the 48 h endpoint fluorescence screening assay were used. Fluorescence values for clones expressing eGFP, as indicated by green fluorescence detection, were normalized by dividing the relative fluorescence units (RFU) by biomass (OD600). The resulting normalized fluorescence values ranged from approximately 300 to 45,000 RFU OD600^−1^, with the majority of tested clones (51%) falling within the range 800 to 1400 RFU OD600^−1^ (Fig. 4A and Supplementary Figure S2). Within this range, the average and median fluorescence for clones with *eGFP* integrations targeting the *ROX1* site was 9–10% lower than those at the *04576* and *PFK1* sites. The highest proportion of clones with very high eGFP fluorescence levels (>8000 RFU OD600^−1^) was obtained for the *04576* integration site (13%), while the lowest proportion (1.4%) was obtained for *PFK1*.

To confirm that clones differed in eGFP fluorescence levels and to evaluate its impact on cell growth, approximately 20 of the fluorescent clones obtained for each integration site were randomly selected for further analysis in a kinetic fluorescence growth assay. Again, a cluster of strains with relatively low fluorescence levels could be seen, in the range 1400 to 1700 RFU OD600^−1^ after 40 h of growth, while a subset of tested clones reached higher expression levels (Fig. 4B and Supplementary Figure S3). The large variation in green fluorescence levels among individual clones suggested that the CRISPR/Cas9 approach resulted in variable *eGFP* expression cassette copy numbers.

Specific growth rates were calculated during the exponential phase for the clones subjected to the kinetic fluorescence growth assay. During the initial phase (from onset of exponential phase to 7.5 h), no significant differences were observed between the specific growth rate of the wild-type control strain, which was 0.13 (SE ± 0.006) h^−1^, and those of the randomly selected fluorescent clones, which ranged from 0.09 (SE ± 0.04) to 0.18 (SE ± 0.01) h^−1^. For subsequent periods, specific growth rates did not differ from the control strain for the majority of clones, with a few exceptions. Namely, a significantly lower growth rate was observed compared with the control strain for two of the *04576* clones and three of the *ROX1* clones (Supplementary Table S1).

These results confirmed that most of the strains with insertions in *04576*, *PFK1* and *ROX1* grew normally on defined DM1-2 medium, using glucose as a carbon source. Thus, in general, the integrated gene cassettes did not affect cell growth and metabolism of the parental strain after gene insertion (Supplementary Figure S4), except for in a limited number of clones.

### Copy number variations assessed by WGS

**Fig. 5:**
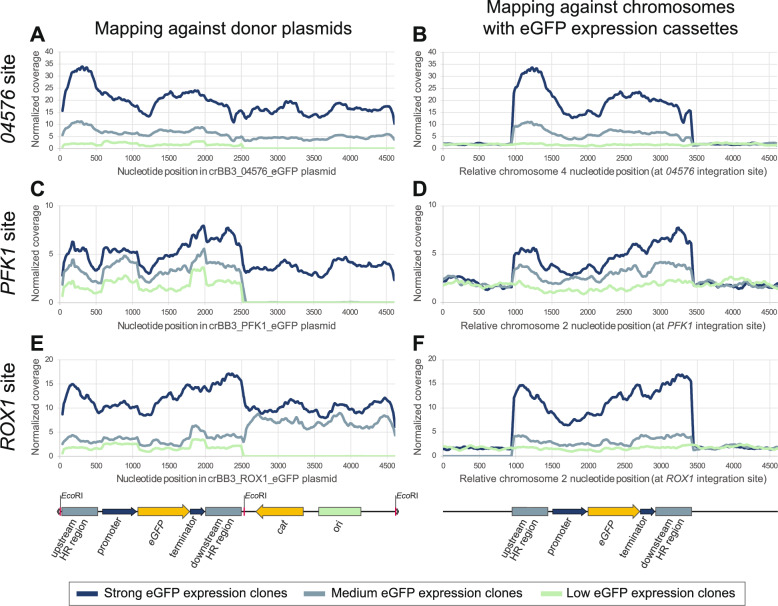
Normalized read depth at each position in **A**, **C**, **E** the plasmids and **B**, **D**, **F** the genomes. The read coverage at each position in the genome and plasmid was normalized by dividing the read coverage at each position by the mean coverage for the entire reference genome. Data are presented as moving averages calculated over 35 nucleotide positions

The differences in fluorescence levels and growth patterns summarized in the previous sections were further explored by means of whole genome sequencing (WGS) analysis. One clone for each of the three integration sites each with low, medium, and strong eGFP fluorescence levels were selected for analysis (labelled with arrows in Fig. 4B). Data on fluorescence levels and growth kinetics for these nine clones are presented in Table 2 and growth curves are shown in Supplementary Figure S4D. Illumina reads were mapped to the *K. phaffii* reference genome sequences [36] modified to contain the expected insertions of *eGFP* expression cassettes, and to the crBB3_04576_eGFP, crBB3_PFK1_eGFP, and crBB3_ROX1_eGFP donor cassette plasmids sequences. Results are shown in Fig. 5A–F and Supplementary Tables S2 to S7, and sequencing and read mapping quality metrics are presented in Table 3.

**Table 2:** Data on fluorescence levels and growth kinetics for isolates subjected to WGS

Strain name	Clone no	Relative fluorescence level	eGFP expression level (RFU OD600^−1^)		Growth kinetics in kinetic plate assay	
			Endpoint assay (48 h)	Kinetic assay (40 h)	µ (h^−1^) 9 to 11 h post inoculation	Max OD600 at stationary phase
*04576*_37	MF8766	low	1173	1588 (SE ± 28)	0.16 (SE ± 0.01)	1.56 (SE ± 0.06)
*04576*_38	MF8765	medium	4754	6479 (SE ± 475)	0.15 (SE ± 0.00)	1.59 (SE ± 0.06)
*04576*_35	MF8764	strong	17913	14955 (SE ± 1 014)	0.13 (SE ± 0.00)	1.65 (SE ± 0.09)
*PFK1*_21	MF8769	low	1335*	1473 (SE ± 55)	0.16 (SE ± 0.00)	1.45 (SE ± 0.05)
*PFK1*_66	MF8768	medium	1726	2277 (SE ± 82)	0.16 (SE ± 0.00)	1.43 (SE ± 0.02)
*PFK1*_72	MF8767	strong	5022	3745 (SE ± 221)	0.15 (SE ± 0.01)	1.32 (SE ± 0.02)
*ROX1*_97	MF8774	low	1019	1492 (SE ± 41)	0.15 (SE ± 0.01)	1.54 (SE ± 0.06)
*ROX1*_87	MF8773	medium	6821	4193 (SE ± 245)	0.12 (SE ± 0.00)	1.39 (SE ± 0.05)
*ROX1*_96	MF8772	strong	7546	8944 (SE ± 293)	0.15 (SE ± 0.00)	1.55 (SE ± 0.05)

*For this clone, the OD600 at 48 h was <1 (0.848)

Further data on growth kinetics is shown in Supplementary Table S1

**Table 3:** Sequencing and read mapping quality metrics for clones subjected to WGS

Strain name	No of PE sequence reads	No of contigs (depth < 5×)	Predicted no of copies of *eGFP*	Read mapping to	Mapped reads	Mean read depth	No of positions with zero read depth
*04576*_37	3,354,227	345	1	Genome	99.08%	54×	34,499
				Plasmid	0.03%	30×	1804
*04576*_38	2,157,059	419	5	Genome	99.11%	39×	35,099
				Plasmid	0.15%	131×	0
*04576*_35	2,506,535	282	14	Genome	98.99%	44×	34,952
				Plasmid	0.49%	250×	0
*PFK1*_21	3,029,252	357	1	Genome	99.11%	48×	36,484
				Plasmid	0.03%	27×	2137
*PFK1*_66	3,928,371	316	2	Genome	99.16%	65×	34,634
				Plasmid	0.05%	70×	1649
*PFK1*_72	3,422,011	328	3	Genome	99.08%	57×	35,164
				Plasmid	0.11%	147×	0
*ROX1*_97	4,626,752	320	1	Genome	99.22%	79×	32,727
				Plasmid	0.03%	47×	1878
*ROX1*_87	902,371	337	2	Genome	98.92%	19×	69,733
				Plasmid	0.14%	60×	0
*ROX1*_96	1,879,340	296	8	Genome	99.09%	37×	32,654
				Plasmid	0.14%	229×	0

Read mapping was performed against both reference genomes and donor cassette plasmids. Also shown are predicted number of *eGFP* copies based on the WGS data. Further data on relative coverages of *eGFP* is shown in Supplementary Tables S2 to S7

### Low fluorescence clones contained single-copy expression cassettes

For the three sequenced clones exhibiting low fluorescence levels (*04576*_37, *PFK1*_21, and *ROX1*_97), BLAST analysis did not indicate the presence of alternative integration sites or multiple insertions. Additionally, PCR analysis covering the entire locus, using primers binding outside of the 5′ and 3′ homologous regions, indicated that each of these low fluorescence clones contained one copy of the *eGFP* expression cassette. The mean normalized mapping read coverage of *eGFP* in the low fluorescence clones, relative to the mean coverage of the entire *K. phaffii* reference genome, ranged from 1.4× to 1.5× (Supplementary Tables S2 to S7). Given that the theoretically expected read coverage of *eGFP* would be 1× for single insertions, these elevated values are likely attributable to uneven read coverages across the chromosomes, a common phenomenon [37]. Therefore, in the remainder of this text, gene read coverages are reported as ‘coverage relative to *eGFP’*, meaning that read coverages are normalized to the mean *eGFP* coverage of the low fluorescence clone for the same target locus (i.e., the coverages of *eGFP* in the low expression clones are set to 1).

Since the promoter and terminator used in the expression cassette were native to *K. phaffii* (both linked to the *TDH3* gene) these sequence fragments were expected to be present twice in clones with single copies of the integrated expression cassettes. When reads were mapped to the donor cassette plasmid references (which contain only one copy each of the *TDH3* promoter and terminator), the mean coverage of the promoter and terminator regions of the expression cassettes was approximately twice as high as the mean *eGFP* coverage; ranging from 1.6× to 1.8× for the promoter and from 2.0× to 2.4× for the terminator (Supplementary Tables S2, S4, and S6). As shown above, the three clones resulted in similar fluorescence levels, ranging from 1473 to 1588 RFU OD600^−1^ after 40 h of growth in the kinetic assay (Fig. 4B and Table 2). Together, these data strongly suggested that these three clones contained one copy of the *eGFP* expression cassette, inserted into the intended target site by CRISPR/Cas9-mediated via homology-directed repair (double crossover cassette exchange).

### Multiple insertions in medium and strong fluorescence clones

BLAST analysis of the *PFK1* medium fluorescence clone (*PFK1*_66) identified one contig covering the junction between two tandem donor cassettes fused in a head-to-tail direction. The 406 bp long contig covered the last part of the downstream homology region, the downstream *Eco*RI site used to linearize the donor helper plasmid, and the first part of the upstream homology region. The mapping read coverages indicated that two copies of *eGFP*, three copies of the *TDH3* promoter and terminator regions, and two copies of each homology region were present (Fig. 5C, D and Supplementary Tables S4 and S5). Sequences matching the remaining parts of the crBB3 donor cassette *E. coli* plasmid backbone, including the cloramphenicol resistance gene (*cat*) and the *E. coli* replication origin (ori), were absent from the genome. These data suggested that two tandem *eGFP* expression cassettes, interspaced by copies of the two homology regions, had been inserted into the *PFK1* target site in the medium fluorescence clone.

The WGS analysis of the *PFK1* strong fluorescence clone (*PFK1*_72) suggested that this genome contained three copies of the *eGFP* expression cassette (Fig. 5C, D and Supplementary Tables S4 and S5). The genome also appeared to contain two or more copies of the plasmid backbone containing *cat* and ori. Contigs were identified that spanned both *Eco*RI sites found on the original crBB3 plasmid. Furthermore, contigs were identified that covered fusions of the donor cassette and the plasmid backbone fragments in the opposite direction than in the original plasmids, i.e., in head-to-head and tail-to-tail configurations. This suggests that multiple fragments had randomly integrated into the target site through ligation of *Eco*RI-generated cohesive ends.

The *PFK1* medium and strong fluorescence clones showed roughly double (2277 RFU OD600^−1^) and triple (3745 RFU OD600^−1^) fluorescence levels compared to the *PFK1* low fluorescence clone (1473 RFU OD600^−1^) after 40 h of growth in the kinetic assay (Fig. 4B and Table 2). This supports the conclusion based on the WGS analysis indicating that these two clones contained two and three copies of the *eGFP* expression cassette.

Copies of the plasmid backbone containing *cat* and ori, as well as unexpected *Eco*RI fusions of *eGFP* donor cassette and the plasmid backbone fragments, were also found in the *04576* and *ROX1* medium and strong fluorescence clones (*04576*_38, *04576*_35, *ROX1*_87, and *ROX1*_96). For example, three clones contained tandem copies of the plasmid backbone fragment fused together in head-to-tail direction at the *Eco*RI site. The number of copies of the donor cassette did not strictly correlate with the number of copies of the plasmid backbone fragments (see e.g. Figure 5E and Supplementary Tables S2, S4, and S6). This further supported the hypothesis that multiple fragments harbouring *Eco*RI cohesive ends had randomly inserted into the target site as part of homology-directed repair after CRISPR/Cas9 cleavage.

The mapping read coverage suggested that the *04576* and *ROX1* medium fluorescence clones (*04576*_38 and *ROX1*_87) contained 5 and 2 copies of the *eGFP* expression cassette, respectively. The corresponding strong fluorescence clones (*04576*_35 and *ROX1*_96) were estimated to have 14 and 8 copies, respectively, based on mapping read coverage, while the fluorescence levels measured in the kinetic assay were approximately 10 and 6 times higher than for the low fluorescence clones (14 955 and 8944 RFU OD600^−1^, respectively; Table 2). It is important to note, however, that these estimates become progressively less precise with increasing copy numbers.

### Loss of the distal chromosomal end downstream of the *ROX1* site

The *ROX1* target site was located on the q arm of chromosome 2, 36.6 kbp from the distal end of the 2.4 Gbp long chromosome. In the *ROX1* medium fluorescence clone the mapping coverage for the region downstream of the integration site (upstream of the upstream homology region) was zero (Fig. 5F), showing that this entire fragment was lost. This corresponds to a loss of 1.5% of the chromosome or 0.4% of the entire genome. In addition to the telomeric region, the missing part of the chromosome contained two rRNA genes and 15 annotated protein coding genes, including *ROX1* itself, which encodes a heme-dependent repressor of hypoxic genes [36, 38].

The loss of the distal chromosomal region was associated with a significantly reduced specific growth rate compared with the control strain. Specifically, during 9–11 h of cultivation, the average growth rate was 0.12 h^−1^, compared to 0.17 h^−1^ for the control strain. Similarly, from 11 to 13 h of cultivation, the average growth rate was 0.11 h^−1^, compared to 0.16 h^−1^ for the control strain (see Supplementary Table S1). At least one other clone out of the 19 *ROX1* clones tested in the kinetic growth assay (*ROX1*_78) showed the same growth pattern as the sequenced clone, as well as a significantly reduced specific growth rate (Supplementary Fig. S4C and Supplementary Table S1).

As seen in Table 2, the fluorescence levels in clones with multiple copies of the *eGFP* expression cassette, relative to that in low fluorescence single insertion clones, were generally lower than predicted based on the copy numbers estimated from the WGS analysis (listed in Table 3). For the *ROX1* medium fluorescence level clone, however, which had two *eGFP* expression cassettes, the RFU OD600^−1^ value was approximately 2.9 times higher than in the low fluorescence clone. The reduced growth rate did not sufficiently explain this discrepancy, as the absolute relative fluorescence was still 2.7 times higher in this clone compared to in the *ROX1* low fluorescence clone.

### Off-target integrations were not observed

There was no evidence in the sequencing data, for any of the nine sequenced clones, suggesting that the *eGFP* expression cassette had inserted into unintended locations on the chromosome. The only fragments containing unexpected fusions were those described above, spanning the *Eco*RI sites of the donor plasmid. Unfortunately, the short read sequencing approach cannot confirm nor disprove the presence of single-crossover insertions of donor plasmids into the *TDH3* promoter, since the 300 bp long reads were shorter than the 493 bp promoter fragment cloned on the donor plasmid. However, no sequence data was identified containing parts of both the *TDH3* gene and any of the downstream homology regions, nor parts of both *eGFP* and the region downstream of the *TDH3* terminator in the wild type genome. This indicates that insertions into the 202 bp long *TDH3* terminator had not occurred.

## Discussion

In this study, we developed a toolkit for CRISPR/Cas9-mediated markerless integration in *K. phaffii*, leveraging Golden Gate cloning and the modular Golden*Pi*CS system [11] for the production of secreted recombinant proteins, and demonstrated its suitability for production of the egg protein ovalbumin. To facilitate the generation of expression clones, donor helper plasmids containing Golden Gate cloning sites flanked by homology regions for the *04576*, *PFK1*, or *ROX1* integration sites, and CRISPR/Cas9 plasmids with sgRNAs targeting each site, were created.

The functionality of each integration site was assessed by analysing clones resulting from targeting each site with an *eGFP* donor cassette. Although it is important to recognize that observed differences may be specific to the properties of the examined expression cassette or expressed protein, and could vary with other inserts or proteins, this analysis revealed several notable differences between the target sites, specifically in (i) transformation efficiency, which was lowest for *04576* and highest for *ROX1* (Fig. 2A); (ii) integration efficiency, which was lowest for *PFK1* and highest for *04576* (Fig. 2B); (iii) eGFP fluorescence levels for single insertion cassette clones, which were lowest for *ROX1* (Fig. 4A); (iv) the frequency of high-expression clones with multiple integration cassettes, which was lowest for *PFK1* and highest for *04576* (Figs. 4 and 5); and (v) the *eGFP* expression cassette copy numbers in the multiple-copy clones, also lowest for *PFK1* and highest for *04576* (Figs. 4 and 5).

While the *04576* site yielded the highest frequency of fluorescent clones (66%), the *ROX1* site resulted in the highest number of transformants, measured as cells transformed with the CRISRP/Cas9 (CRIS*Pi*) plasmid (~2000 cfu µg^−1^ DNA). The transformation efficiency for *ROX1* was of the same order of magnitude as that observed in a previous study using wild-type *K. phaffii* and similar amounts of CRISPR/Cas9 plasmid and donor cassettes as in the current study [20]. The efficiency of integration following transformation of *K. phaffii* is known to vary depending on the integration site, at least in the case of integrative vectors [39], and a trade-off between transformation and targeting efficiencies similar to that observed in the current study has been previously observed with CRISPR/Cas9 systems. This effect has been linked to differences in the sgRNA sequences, as highly efficient sgRNAs can lead to increased rates of double strand breaks, which, if not efficiently repaired, can reduce transformation efficiency [20, 40, 41]. Furthermore, we observed an increase in the integration efficiency with a longer recovery time after electroporation (1 vs. 3 h), which aligns with previous findings [26], suggesting that homologous recombination-based repair is time-dependent. The increased number of transformants after longer incubation was likely due to transformant replication. This was evident in the *ROX1* reactions, where transformation efficiency doubled from 1 to 2 h of incubation, while the percentage of green fluorescent clones remained the same.

The donor cassette integration efficiencies reported in the current study, ranging from 20 to 70%, were comparable to those achieved with a *KU70* deletion mutant, which is deficient in non-homologous-end-joining (NHEJ) repair [27, 40, 42]. Zhou et al. [43] also reported comparable integration efficiencies for two other *K. phaffii* strains deficient in NHEJ repair, a *KU70* deletion mutant and a *DNL4* deletion mutant. Integration efficiencies in similar ranges were shown for *K. phaffii* strains constructed for enhanced HR efficiency by Cai et al. [26], while higher values were reported by Gao et al. [44]. Although further enhancement of the HR machinery could lead to higher integration efficiency, the obtained rates in the present work were sufficient for producing clones without the need to screen an excessive number of transformants. Notably, this was accomplished without the associated drawbacks of *KU70* deletion, such as increased sensitivity to DNA damage and potential stability issues [42]. Other yeast systems have shown similar or slightly higher efficiencies after being subject to optimised CRISPR/Cas9-mediated integration, such as *S. cerevisiae* [45, 46] and the nonconventional yeast *Kluyveromyces marxianus* [47].

It is well-known that the protein expression levels in *K. phaffii* may vary depending on the integration locus [25, 27]. In this study, fluorescence levels were found to be 9–10% lower when *eGFP* was targeted to the *ROX1* integration site compared to *04576* and *PFK1*. This moderate effect aligns with previous work showing that gene copy number had a much greater influence on expression than the integration locus [48]. It should, however, be noted that the three target sites used in this study were all previously identified as functional sites [21], which may explain why the fluorescence levels were relatively similar. However, since *PFK1* and *ROX1* are located on chromosome 2 and *04576* on chromosome 4, the results contradict earlier findings that expression levels decrease with increasing chromosome number and length [25].

WGS of selected clones revealed the insertion of variable copy numbers of *eGFP* expression cassettes, which explained variations in fluorescence between clones. Insertion of plasmid backbone fragments at the target sites was also identified, but off-target integrations were not observed. The observed disproportionate correlation of estimated eGFP gene copy numbers estimated from WGS data and fluorescence levels suggested that fluorescence levels in high copy number clones may be limited by other factors, such as the efficiency of the expression or secretion machinery. Similar observations have been made also using classical *K. phaffii* expression systems [49].

WGS analysis revealed that multiple donor cassettes and plasmid backbone fragments were stitched together through the *Eco*RI-generated cohesive ends generated during linearization of the donor helper plasmid. This likely occurred through in vivo ligation, either before or during chromosomal integration via homology-directed repair at the target sites. In vivo ligation has been previously described both in *K. phaffii* [18] and *S. cerevisiae* [50]. Multi-copy gene insertions present pitfalls such as clone instability, unpredictability and the lack of a linear relationship between number of gene insertions and increase in product [51]. To address the above-mentioned finding and promote single copy insertions, the DNA can be treated prior to transformation to eliminate the cohesive ends, e.g., using T4 DNA Polymerase. To further prevent plasmid backbone insertion, the donor cassette can be gel-purified or amplified using PCR. The phenomenon was observed across different integration sites, indicating a consistent issue with the current methodology. However, the *04576* site showed the greatest number of both high copy number insertion clones and number of inserts per clone, whereas the *PFK1* site had the lowest in both cases. This pattern reflected the differences in integration efficiency, with *04576* having the highest efficiency and *PFK1* the lowest. The underlying mechanisms behind this co-occurrence and the reasons for this correlation remain unclear. Potentially, it could be related to the potency of the sgRNA or differential accessibility of the cell’s repair machinery at the three integration sites [52]. Further conclusions on the cause behind these findings could be achieved through comparing integration efficiency between different sgRNAs targeted to the same integration site.

Previous studies have reported the presence of chromosomal arrangements, large deletions, and other structural variants during generation of mutants in *K. phaffii* [18, 53] and other eukaryotic cells [54, 55]. In one of the three *ROX1* clones sequenced in the current study, loss of the distal 36.6 kbp of the chromosome was observed after gene insertion. The loss of the tip of the chromosome end in the sequenced clone coincided with a significantly reduced specific growth rate, and two more of the 19 *ROX1* clones tested in the kinetic growth assay presented similar growth patterns, suggesting that similar issues were present also for other clones. However, this hypothesis is challenging to confirm with short-read WGS data alone. For more detailed insights into potential chromosomal rearrangements, other approaches such as long read sequencing could be employed [56]. Further work should be undertaken to identify strategies to mitigate unwanted CRISPR/Cas9 effects, such as larger chromosomal modifications and deletions, in CRISPR/Cas9 applications. It is essential to check for said unwanted effects to avoid selecting clones with such issues for downstream work.

The suitability of the present method to produce proteins of interest for the food industry was proven by generating clones expressing and secreting recombinant chicken egg ovalbumin. An anticipatory life cycle assessment published in 2021 indicated that production of ovalbumin by means of precision fermentation and cellular agriculture can have a lower impact than traditional agriculture approaches [57]. *K. phaffii* is a relevant industrial host for recombinant ovalbumin production, as evidenced by the presence of a company in the market leveraging this strategy [4, 58, 59]. To the best of our knowledge, the current work is the first report of CRISPR/Cas9 applied to produce recombinant chicken ovalbumin using microorganisms.

Based on the molecular size observed on SDS-PAGE, two variants of ovalbumin were secreted by the clones generated in this work: a lower molecular weight species and a higher molecular weight species, with apparent sizes of 45 and 47 kDa, respectively. Given that both variants were immunoreactive with chicken ovalbumin antibodies, the presence of two fractions may be explained by different glycosylation patterns. Previous studies have reported two components with similar molecular weights and relative production ratios using yeasts to produce recombinant chicken ovalbumin [34, 35, 59]. Site-specific glycosylation plays an important role in protein folding and secretion during recombinant protein production in *K. phaffii* [60]. In the past years, methods have been developed to modify the glycosylation of proteins heterologously produced in *K. phaffii* [61, 62]. This approach, known as glycoengineering, can alter the characteristics and improve the properties of the recombinant products. Glycoengineering could be explored as a mean to modify the functional properties and/or to reduce the antigenicity of native egg ovalbumin, as suggested in previous studies [63, 64].

The average total titers of secreted ovalbumin observed in the current work were 35 mg L^−1^. Slightly lower values (10 mg L^−1^) have been reported in previous studies using the same host species, promoter and protein of interest [34]. Given the relevance of ovalbumin for the food industry, other host species have been explored to recombinantly produce this protein. In this regard, the yeast *S. cerevisiae* was able to produce 132 mg L^−1^ in a glucose-limited fed-batch fermentation process, after optimization of promoter and signal peptide and co-expression of helper proteins; however, the secretion capacity of the constructed strain was limited, as only 8 mg L^−1^ of the recombinant ovalbumin was extracellular [35]. Similar values were initially observed in another study heterologously producing chicken ovalbumin in *S. cerevisiae*, where initial reported titers were 3.4 mg L^−1^, rising to 166.3 mg L^−1^ after the co-expression of helper proteins, optimization of expression levels of several regulators, and modification of bioreactor cultivation conditions [65]. *K. phaffii* and the *AOX1* promoter have been used to produce recombinant ovalbumin from Chinese quail oviduct, obtaining accumulated productions after 48 h of cultivation ranging from 450 to 5450 mg L^−1^, with great differences between clones, presumably due to gene dosage effects [66]. Furthermore, other eukaryotic expression hosts have been successfully used to produce recombinant chicken ovalbumin, such as the filamentous fungi *Trichoderma reseei*, which was able to secrete up to 2 g L^−1^ in fed-batch bioreactor cultivations [67]. The recombinant ovalbumin production levels observed in the present work could be increased through different approaches, such as the use of more efficient promoters [68–70], or employing a host strain that shows more favourable protein folding, secretion, or general improved cell fitness [71]. The current expression strain can also be modified through metabolic engineering to incorporate these (or other) genetic enhancements [17]. Finally, improvements may be implemented at the bioprocess level, modifying the cultivation parameters to further increase production efficiency [70, 72, 73].

## Conclusions

Our approach aimed to address several challenges associated with heterologous gene expression in *K. phaffii*, including a deeper understanding of the dynamics of CRISPR/Cas9 systems for strain engineering, the need for efficient and precise genome integration, the identification of optimal integration sites for expression of proteins, and convenient and time-efficient cloning procedures for generation of clones with different expression cassette design. Further extension of the toolkit can be generated by including additional integration sites, as well as assembling a library of secretion signal sequences.

Overall, our study demonstrates the feasibility and effectiveness of combining CRISPR/Cas9 technology with modular cloning systems for the precise and efficient production of recombinant proteins in *K. phaffii*. This approach holds promise for expanding the range of high-value proteins that can be produced using microbial cell factories. Future research should focus on addressing the identified challenges to fully realize the potential of this technology, including optimizing integration sites and improving cloning procedures to enhance the system’s efficiency and versatility.

## Materials and methods

### Strains and cultivation

*Escherichia coli* NEB 10-beta Competent *E. coli* (New England Biolabs) were used for plasmid construction. *E. coli* was cultured in tryptone soya broth (TSB; Oxoid), plated on 1.5% TSB agar (Oxoid), and grown at 37 °C. For selection in *E. coli*, 50 µg mL^−1^ kanamycin sulfate, 100 µg mL^−1^ ampicillin, or 25 µg mL^−1^ chloramphenicol (all from Sigma-Aldrich) were used.

The *K. phaffii* strain used was ordered from ATCC through LGC Standards as *Komagataella phaffii* Kurtzman (ATCC 76273, NRRL Y-11430, CBS 7435). *K. phaffii* was routinely cultured in YPD broth (BD Difco from Fisher Scientific) or on 1.5% YPD agar plates (Oxoid). Agar plates were incubated at 30 °C. Submerged cultures were grown in 500 mL baffled Erlenmeyer flasks (Bellco Biotechnology). Geneticin (G418; Sigma-Aldrich) at 500 μg mL^−1^ was used for selection of CRIS*Pi* plasmids.

The DM1-2 defined medium, used for kinetic growth assays, is a modification of the DM1 medium [74] containing 1.7 g L^−1^ Yeast Nitrogen Base, 7 g L^−1^ urea, 10 g L^−1^ KH_2_PO_4_ pH 6.5, 22 g L^−1^ D(+)-Glucose·H_2_O (all from Sigma-Aldrich), 1× Gibco MEM amino acids solution, 1× Gibco Cholesterol Lipid Concentrate (both from ThermoFisher Scientific) and 100 µL L^−1^ PTM1s trace mineral solution. PTM1s was prepared by dissolving 0.2 g L^−1^ biotin, 0.02 g L^−1^ boric acid, 0.92 g L^−1^ CoCl_2_·6H_2_O, 6 g L^−1^ CuSO_4_·5H_2_O, 65 g L^−1^ FeSO_4_·7H_2_O, 3 g L^−1^ MnSO_4_·H_2_O, 0.08 g L^−1^ NaI, 0.2 g L^−1^ Na_2_MoO_4_·2H_2_O, 20 g L^−1^ ZnCl_2_, 5 mL L^−1^ H_2_SO_4_ (all from Sigma-Aldrich) in milliQ-H_2_O and subsequent sterile filtration.

### Molecular biology procedures

Primers, genes, and gene strands were ordered from Eurofins Genomics. Restriction enzymes and ATP were from New England Biolabs. Plasmids were isolated using the QIAprep Spin Miniprep kit (Qiagen). Templates for PCR of *E. coli* clones were generated by transferring small samples from single colonies to PCR tubes followed by heating for 1 min at 800 W in a microwave oven. Templates for PCR of *K. phaffii* clones were generated by resuspending small samples of single colonies in 20 µL 0.1 M NaOH, incubating for 25 min at 99 °C in a ThermoCycler, and using 1 µL in each PCR reaction.

Routine verification of plasmids and strains was performed by PCR of the targeted loci using Platinum HotStart PCR Master Mix (2X) (Invitrogen) and 0.2 µM each of forward and reverse primers. PCR primers with annealing sites located outside of the integration sites on plasmids are listed in Supplementary Table S8. Sequencing of plasmid constructs was performed using Sanger sequencing with the BigDye Terminator v3.1 Cycle Sequencing Kit and the Genetic Analyzer 3500 instrument (both Applied Biosystems). PCR screening of OVA transformants and eGFP expression clones subjected to WGS was performed with primers covering the 5′ site of the targeted sequence to detect transformants and primers covering the entire locus (binding outside of the 5′ and 3′ homologous regions) to identify wild-type clones. The PCR primers are listed in Supplementary Table S8.

Plasmids utilized in the present work were constructed by means of Golden Gate assembly. The Type IIS restriction endonuclease *Bbs*I, which has identical recognition and cleavage specificities as *Bpi*I, was used in the current study to generate ssDNA sticky ends. *Bbs*I Golden Gate assembly reactions were performed using 0.5 µL *Bbs*I-HF (10 U), 0.1 µL T4 ligase (40 U), 2 µL rCutSmart Buffer (10×), 2 µL ATP (10 mM) in 20 µL volumes. *Bsa*I Golden Gate assembly reactions were similarly performed using 1 µL NEB Golden Gate Assembly Mix (*Bsa*I-HFv2) and 2 µL T4 DNA Ligase Buffer (10×). Reactions used ~50 fmol vector and ~100 fmol of each insert and were incubated for 30 cycles of 5 min each at 37 °C and 16 °C, followed by heat inactivation for 6 min at 60 °C, except for construction of the ovalbumin expression cassette, where ~10 fmol vector and ~50 fmol of each insert was used, and the reaction was incubated for 100 cycles of 5 min at 37 °C and 3 min at 16 °C. Further details on the specific Golden Gate assembly reactions used to construct the plasmids employed in the present work can be found in subsection “Plasmid construction” below.

Plasmids containing donor cassettes were linearized prior to transformation into *K. phaffii* by digestion of 5 µg plasmid with 5 µL *Eco*RI-HF in 250 µL reaction volumes. Complete digestion was confirmed by agarose gel electrophoresis, the DNA was concentrated by ethanol precipitation with 0.1× volumes of 3 M Na-acetate (pH 4.8–5.2), dissolved in 10 µL dH_2_O, and the DNA concentration determined using NanoDrop.

### Plasmid construction

#### CRIS*Pi* sgRNA/Cas9 plasmids

CRISPR/Cas9 mediated gene editing was performed using plasmids constructed from a vector with functional Cas9 and a sgRNA expression cassette insertion site included in the CRIS*Pi* Kit [22], which was a gift from Brigitte Gasser (Addgene Kit #1000000136). The sgRNA/Cas9 plasmids were constructed using the BB3cK_pGAP_23*_pLAT1_Cas9 (CRIS*Pi*) plasmid, in which expression of *cas9* is under the control of the *LAT1* promoter and the *kanMX* selection marker is used for positive selection in *E. coli* (kanamycin) and *K. phaffii* (G418). The sgRNA target sequences used were obtained from previous work [21] and are listed in Supplementary Table S9. The construction of the HH-sgRNA-HDV fusion gene and its insertion into the CRIS*Pi* plasmid was performed essentially as described elsewhere [22], but with an alternative strategy for the overlap extension (OE) PCRs. The invariant region was covered by one long reverse oligo (sgRNA_struc_rev), while the target-specific sgRNA regions were covered by long forward oligos partly overlapping with the reverse oligo. In addition, short primers containing the 18–20 first nucleotides of each of the long primers were designed (Supplementary Table S10). The PCRs were run using 0.01 µM of each long oligo, 0.5 µM of each short primer, 200 µM dNTPs, and Q5 Hot Start High-Fidelity DNA Polymerase (New England Biolabs) using 30 cycles of 98 °C/10 s, 55 °C/20 s, and 72 °C/10 s. The *04576* sgRNA fragment was precloned into the Invitrogen pCR4Blunt-TOPO vector (Thermo Fisher Scientific), while the PCR products for the *PFK1* and *ROX1* sgRNA fragments were purified from a 2% NuSieve GTG agarose gel (Lonza), prior to *Bbs*I (*Bpi*I) Golden Gate assembly cloning into the CRIS*Pi* plasmid. Reactions were transformed into *E. coli* and selected on kanamycin, to generate plasmids CRIS*Pi*_04576, CRIS*Pi*_PFK1, and CRIS*Pi* _ROX1 (Table 1).

#### crBB3 donor helper plasmids

The pGGAselect plasmid from the *Bsa*I-HFv2 NEB Golden Gate Assembly Kit was used as the backbone for generating the crBB3 plasmid vectors. Approximately 500 bp sequences for homologous recombination, located upstream and downstream of the CRISPR/Cas9 target sites near the *04576*, *PFK1* and *ROX1* loci (i.e., between genes BQ9382_C4-3965 and BQ9382_C4-3970 on chromosome 4, in BQ9382_C2-4830 on chromosome 2, and upstream of BQ9382_C2-6940 on chromosome 2, respectively, in the *K. phaffii* CBS 7435 reference sequence), were either generated by PCR using Q5 Hot Start High-Fidelity DNA Polymerase and precloned into the pCR4Blunt-TOPO vector (for *04576* downstream) or ordered as synthesized genes (Supplementary Table S11). *Bpi*I, *Bsa*I, and *Eco*RI restriction sites were removed by introducing point mutations in the synthesized sequence fragments. The Fs1–Fs4 linker fragment, to be inserted between the upstream and downstream homology regions, contained *Bpi*I restriction sites and Fs1 and Fs4 fusion sites for cloning of transcription units, preceded by a strong artificial transcriptional terminator to prevent potential transcriptional read-through from the upstream gene after insertion of the expression unit in the *K. phaffii* genome. The ends of all fragments were designed to contain *Bsa*I restriction sites and appropriate fusion sites, which were used to combine all three fragments in pGGAselect using *Bsa*I Golden Gate assembly. Reactions were transformed into *E. coli* and selected on chloramphenicol, generating plasmids crBB3_14_04576, crBB3_14_PFK1, and crBB3_14_ROX1 (Table 1 and Supplementary Figure S5). Plasmids crBB3_AC_04576, crBB3_AC_PFK1, and crBB3_AC_ROX1 were generated by replacing the Fs1-Fs4 linker with one containing *Bsa*I restriction sites and fusion sites FsA and FsC (Supplementary Table S11), using *Bbs*I (*Bpi*I) Golden Gate assembly reactions.

Note: The *PFK1* upstream homology region contains a poly(dA·dT) sequence consisting of 10 thymine (T) bases. When performing PCR over this region prior to Sanger sequencing, DNA polymerase slippage can occur, which may lead to a mixed population of PCR products, complicating the sequencing results. This issue does not arise when sequencing is performed using a plasmid miniprep as template.

#### *eGFP* and *OVA* expression cassette plasmids

The Golden*Pi*CS Kit [11] was a gift from the Gasser/Mattanovich/Sauer group (Addgene kit #1000000133). Plasmids BB1_12_pGAP, BB1_23_eGFP, and BB1_34_TDH3tt from Golden*Pi*CS were used to assemble eGFP donor cassettes for homologous recombination by Golden Gate assembly with *Bbs*I (*Bpi*I) and donor helper plasmids crBB3_14_04576, crBB3_14_PFK1, and crBB3_14_ROX1. *GAP* and *TDH3* are synonyms for the gene encoding glyceraldehyde-3-phosphate dehydrogenase (isozyme 3), with locus tag BQ9382_C2-4615 in the *K. phaffii* reference genome [36, 75].

The *S. cerevisiae* α-mating factor (α-MF) sequence from the pPIC9 vector (Invitrogen) with flanking sequences for Golden Gate assembly and with replacement of the *Xho*I restriction site within the coding region to TTAGAG (conservative substitution) was ordered as a synthetic dsDNA fragment (Supplementary Table S12) cloned into the vector pEX-A128 (Amp^R^), generating pEX-A128-αMF (Table 1). The upstream fusion site (Fs) constitutes T (from the flanking sequence) plus the α-MF ATG start codon (TATG). The downstream fusion site constitutes the last four bases of the α-MF coding sequence (AGCT, identical to Golden*Pi*CS FsD and referred to as FsX in Fig. 3). This plasmid was used as a template in a PCR reaction to change the 5′ fusion site from TATG to CATG (Fs2) using primers aMF-fwd-Fs2 and aMF-rev-FsD (Supplementary Table S12) and Q5 Hot Start High-Fidelity DNA Polymerase, enabling seamless fusion between the promoter modules from the Golden *Pi*CS kit and the α-MF signal sequence. The product was cloned into the pCR4Blunt-TOPO vector, transformed into *E. coli* and selected on ampicillin, generating plasmid pTOPO-αMF (Table 1). This plasmid carries both kanamycin and ampicillin selection markers, thus the ampicillin resistance gene needs to be removed if pTOPO-αMF is to be used as a BB1 module during assembly of more than one transcription unit into BB2 backbone vectors (but does not affect cloning directly into BB3 vectors).

The *OVA* gene (NBCI accession AH002466) from *Gallus gallus* (chicken) encoding ovalbumin protein (accession P01012), excluding the initial methionine codon, was codon-optimised using the *K. phaffii* codon usage frequencies in the Kazusa database [76] and designed without *Bpi*I, *Bsa*I, *Eco*RI, *Xho*I, *Bam*HI, nor *Not*I restriction sites. Flanking sequences containing *Bpi*I restriction sites and appropriate fusion sites were included and the fragment was ordered as a synthetic dsDNA fragment (Supplementary Table S12) cloned into the pEX-K248 vector, generating pEX-K248-OVA (Table 1). Plasmids BB1_12_pGAP and BB1_34_TDH3tt from the Golden*Pi*CS toolkit [11], pTOPO-αMF, and pEX-K248-OVA were subjected to Golden Gate assembly with *Bbs*I (*Bpi*I) and the crBB3_14_04576 donor helper plasmid, generating plasmid crBB3_04576_OVA (Table 1 and Supplementary Figure S5).

Reactions were transformed into *E. coli* and selected on chloramphenicol, generating plasmids with *eGFP* or *OVA* donor cassettes for homology-directed repair. Plasmids were linearized with *Eco*RI prior to transformation as described.

### Transformation of *K. phaffii*

Electrocompetent *K. phaffii* cells were prepared essentially as described [39]. Cultures were inoculated at 0.1% (v/v) from glycerol stocks (15% glycerol) prepared from exponential phase cultures and grown at 28 °C with shaking at 120 rpm to optical density at 600 nm (OD600) between 1.2 and 1.5 (~10^7^ cells mL^−1^). Cells were centrifuged at 500 *g* for 5 min at room temperature, gently resuspended in 25 mL freshly made room-temperature sterile-filtered resuspension buffer (100 mM lithium acetate, 10 mM dithiothreitol, 0.6 M sorbitol, and 10 mM Tris–HCl, pH 7.5), and incubated at 20 °C with gentle mixing at 100 rpm for 20–30 min. Cells were centrifuged as before, gently resuspended in 750 µL ice-cold 1 M sorbitol, and transferred to 2-mL microtubes placed on ice. They were then centrifuged at 4 °C and washed twice with 750 µL ice-cold 1 M sorbitol, before final addition of 50 µL ice-cold 1 M sorbitol and gentle resuspension. Aliquots of 80 µL, kept on ice, were used the same day.

Electrocompetent *K. phaffii* cells were co-transformed with 100 ng CRIS*Pi* plasmid and linearized crBB3 donor plasmid, corresponding to 1.0 or 1.1 µg donor cassette DNA. Cells were electroporated in 0.2-cm gap cuvettes (Bio-Rad) at 1.5 kV, 25 µF, and 200 Ω in a MicroPulser (Bio-Rad). 1 mL ice-cold YPD medium diluted 1:1 in 1 M sorbitol was added to the cells immediately after electroporation. Cells were allowed to regenerate for 1–3 h at 28 °C with gentle shaking at 100 rpm before selecting on YPD agar containing G418 to select for transformants harbouring CRIS*Pi* plasmids. At each time-point, 300 µL was plated on a total of six agar plates. Transformants were counted after 4 to 7 days of incubation. The CRIS*Pi* plasmid (containing ARS replication region) was cured by growth on non-selective media. For transformation with the *eGFP* donor cassettes, three independent experiments (biological replicates) were performed.

### Fluorescence assays

For the endpoint fluorescence screening assay, up to 100 single colonies from each *eGFP* cassette transformation reaction and plating time-point (picked at random) were used to inoculate 200 µL of YPD cultures in black clear bottom 96-well plates (Greiner Bio-One) with lids. For each reaction and time-point, all clones were picked if fewer than 100 colonies were present; otherwise, 100 clones were selected. Plates were incubated with shaking at 28 °C and 180 rpm for 48 h. Measurements of OD600 (to confirm growth) and fluorescence at 484 nm excitation and 510 nm emission, read from the plate bottom, were obtained in a Synergy H1M instrument (BioTek Instruments / Agilent Technologies).

Targeting efficiency was calculated as the percentage of fluorescent clones above a threshold set by the readings obtained for negative controls (relative fluorescence units [RFU] above 600, obtained with gain set to 50). For evaluation of normalized fluorescence (RFU OD600^−1^), only clones with OD600 > 1 were included.

For the kinetic fluorescence growth assay, seed cultures were prepared by inoculating single colonies from YPD agar plates containing G418 into 5 mL YPD and incubating overnight at 28 °C and 200 rpm. Overnight seed cultures were diluted to 0.5% (v/v) in 200 µL DM1-2 defined medium in 96-well black clear bottom plates (Greiner Bio-One) with lids. The side of plates with lids was covered with parafilm to minimize volume losses due to evaporation and incubated in the Synergy H1M instrument at 28 °C, with slow-speed 1 mm-amplitude orbital mode mixing (10 Hz) at 559 cpm. OD600 and RFU were measured every 5 min as described above. Two to four biological replicates were used for each transformant. Growth and fluorescence data were represented using the python libraries Matplotlib v3.9.3 [77], NumPy v2.2.0 [78] and Pandas v2.2.3 [79].

### Statistical analyses

Statistical differences in transformation and integration efficiencies were evaluated using Minitab v.22 software and three-way analysis of variance (ANOVA) with integration site, sampling time-point, and biological replicate as factors. The tested null hypotheses were no differences between integration sites and no differences between time-points. After rejection of a null hypothesis (*p* < 0.05), Tukey’s post hoc test for pairwise comparisons was performed to compare differences between groups. The full results for this statistical analysis is shown in Supplementary Tables S13–S16.

Statistical analyses for the kinetic fluorescence growth assay were performed in GraphPad Prism 10.3.1. A significance level of *p* = 0.05 was employed to evaluate differences in group means. When the data had normal distribution and homoscedastic variance, ordinary one-way ANOVA was applied, followed by Dunnett’s test. Kruskal–Wallis test (one-way ANOVA on ranks) followed by Dunnett’s T3 test was used when the data was not normally distributed. The full results for the ANOVA analysis is shown in Supplementary Tables S17–S19.

### Analysis of ovalbumin expression

For production of recombinant ovalbumin in *K. phaffii*, overnight 50 mL YPD seed cultures from single colonies were used to inoculate 100 mL of DM1-2 defined medium in 500 mL baffled Erlenmeyer flasks covered with cotton plugs, to obtain a starting OD600 of 0.005. Cultures were grown at 25 °C and 200 rpm for 48 h before supernatants were harvested by centrifugation at 17000 *g* for 4 min at 4 °C and snap-frozen on liquid N_2_.

Supernatant samples or standard protein samples (albumin from chicken egg white; Sigma-Aldrich) were mixed with Invitrogen Bolt LDS Sample buffer and 100 mM DTT, and heated at 70 °C for 10 min before loading onto SDS-PAGE gels; 12-well Invitrogen Bolt Bis–Tris Plus mini protein gels, 4–12%, 1.0 mm, WedgeWell format. Invitrogen BenchMark pre-stained protein ladder (10 µL; ThermoFisher Scientific) and Amersham ECL Plex fluorescent Rainbow marker (5 µL; Cytiva) were used as molecular weight markers for total protein staining and Western blot, respectively. Gels were run in Invitrogen Bolt MOPS SDS running buffer for 40 min at 200 V. For total protein staining, gels were washed for 3 × 5 min with milliQ-H_2_O, incubated for 1 h with Invitrogen SimplyBlue SafeStain (all from ThermoFisher Scientific) and washed with milliQ-H_2_O overnight before imaging in a Perfection 4990 Photo scanner (Epson).

For western blotting, SDS-PAGE gels were blotted onto iBlot 3 nitrocellulose membranes using the Invitrogen iBlot 3 transfer system (both from ThermoFisher Scientific). After transfer, membranes were blocked in TBS-Tween with 2% ECL Prime blocking agent (Cytiva) for 1 h, rinsed twice with TBS-Tween and then incubated overnight at 4 °C with primary antibody (rabbit anti-OVAL antibody; Sigma-Aldrich) diluted 1:2000 in TBS-Tween with 0.2% ECL Prime blocking agent. Membranes were subsequently washed three times for 5 min with TBS-Tween, incubated for 1 h at room temperature with secondary antibody (Amersham ECL Plex Cy5 conjugated goat-anti-mouse IgG; Cytiva) diluted 1:2000 in TBS-Tween with 0.2% ECL Prime blocking agent, and finally washed three times for 5 min with TBS-Tween. Membranes were air-dried before protein bands were visualized in a G-Box gel-doc system (Syngene). Densitometric analysis of the images was performed with the ImageQuantTL software (Cytiva).

### Genome sequencing and analysis

For preparation of genomic DNA for Illumina sequencing, cells were lysed using Lysing Matrix B and a FastPrep instrument (both MP Biomedicals) and DNA isolated using the DNeasy Blood and Tissue Kit (Qiagen). Libraries for genome sequencing were prepared using the Nextera XT DNA Sample Preparation Kit (Illumina) and sequenced using paired-end (PE) 2× 300 bp reads on a MiSeq instrument (Illumina).

The reference genomes used for mapping were the four chromosomes contained in the *K. phaffii* reference genome sequence [36] (*Komagataella phaffii* CBS 7435 NCBI GenBank assembly GCA_900235035.2), manually modified to contain the expected insertions of each of the three *eGFP* expression cassettes, in addition to the sequences of the *K. phaffii* CBS 7435 mitochondrion (GenBank accession NC_015384.1) and the two killer plasmids (GenBank accessions MG491503.1 [13.1 kb plasmid] and MG491504.1 [9.5 kb plasmid]).

Raw reads were filtered on q15 and trimmed of adaptors before they were mapped to the modified versions of the *K. phaffii* reference genome (described above) and to the sequences of the crBB3_04576_eGFP, crBB3_PFK1_eGFP, and crBB3_ROX1_eGFP donor cassette plasmids (Table 1) using Burrows–Wheeler Aligner (BWA-MEM) v0.7.15 [80]. The alignment was converted to BAM format, sorted, indexed, and duplicates were marked using SAMtools v1.10 [81] and Picard v2.22.3 [82].

The percentage of mapped reads was determined using SAMtools flagstat. The mean read depth coverage of the entire *K. phaffii* reference genome and the number of positions on each reference with zero coverage was calculated using Picard CollectWgsMetrics. The read depth coverage at each position in each reference was obtained by SAMtools depth (with option -a). Normalized depth of coverage at each position in each reference was obtained by dividing the read depth coverage at each position by the mean read depth coverage for the entire *K. phaffii* reference genome. ‘Mean normalized coverage’ was calculated as the mean normalized depth of coverage for a specific gene or genetic region. ‘Coverage relative to *eGFP*’ was calculated by dividing the mean normalized coverage of a specific gene or DNA fragment by the mean normalized coverage of the *eGFP* gene in the low fluorescence single gene insertion clone, for the corresponding target site (Supplementary Tables S2 to S7).

Genome assembly was performed with SPAdes v3.13.0 [83] and six *k*-mer sizes (21, 33, 55, 77, 99, and 127). Sequences from the expected integration regions, the region surrounding the *TDH3* gene, and the crBB3 donor cassette plasmids were used as queries in BLAST (blastn v2.12.0+) to search for contigs with alternative integration sites or multiple insertions.

## Supplementary Information

Additional file 1.

Additional file 2.

## Data Availability

Maps and GenBank sequences of key plasmids generated in this study are provided in the Supplementary Information files. The plasmids obtained during the study are available from the corresponding author on reasonable request. The raw Illumina sequencing reads have been deposited to the National Center for Biotechnology Information (NCBI) Sequence Read Archive (SRA) under BioProject number PRJNA1188001.
