# Supplementary material for: A toolkit for facilitating markerless integration of expression cassettes in Komagataella phaffii via CRISPR/Cas9
Source: Microb Cell Fact. 2025 May 3;24:97. doi: 10.1186/s12934-025-02716-x (PMC12049782; doi:10.1186/s12934-025-02716-x)
Supplement: Supplementary file 1 — Additional file 1. [file 12934_2025_2716_MOESM1_ESM.pdf]

Supplementary Information

Supplementary File S1

A toolkit for facilitating markerless integration of expression cassettes in *Komagataella phaffii* via CRISPR/Cas9

Laura García-Calvo, Charlotte Kummen, Solvor Rustad, Sissel Beate Rønning, Annette Fagerlund

Nofima – Norwegian Institute of Food, Fisheries and Aquaculture Research, Ås, Norway

Microbial Cell Factories, 2025

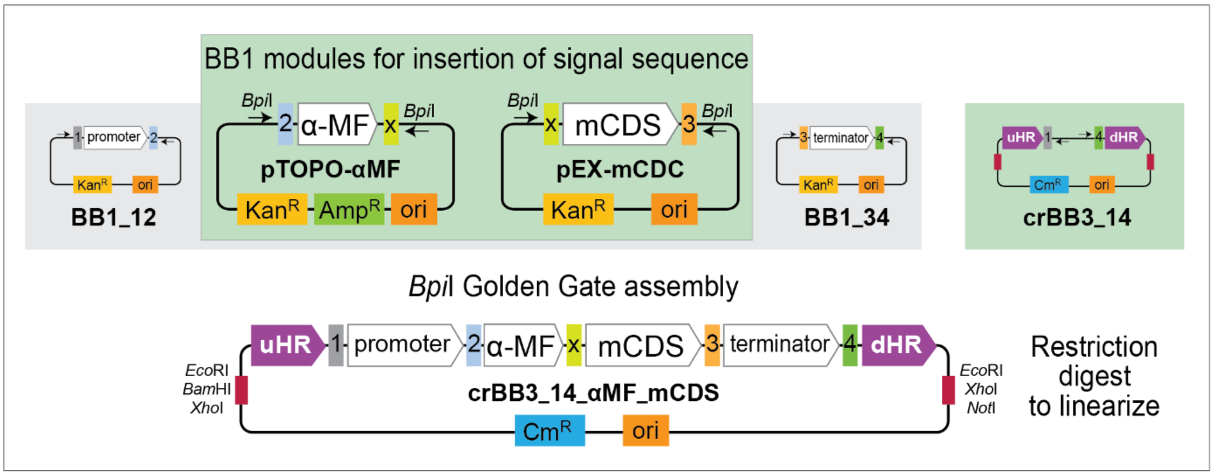

Supplementary Figure S1: Construction of donor expression cassettes for secreted proteins, as used for *OVA* in the current study. Shown is a construct carrying the α-MF secretion signal sequence. The fusion site labelled X linking the secretion signal with the mature coding sequence should be chosen to match the last 4 bases of the secretion signal or the first 4 bases of the coding gene. For use in generation of multiple expression cassettes on the crBB3_AC plasmid (or BB3 level vectors from Golden*Pi*CS), the ampicillin resistance marker on the vector carrying the α-MF secretion signal sequence must be removed.

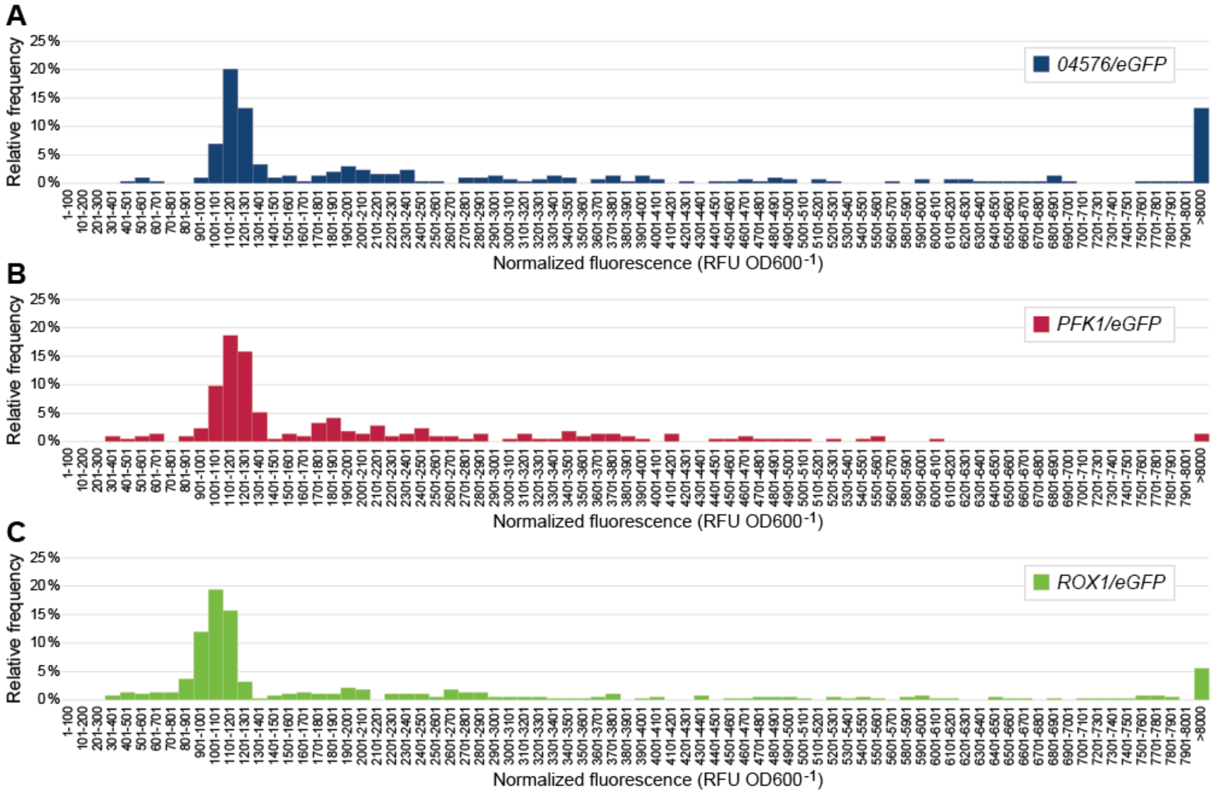

Supplementary Figure S2: Distribution of fluorescence levels determined by an endpoint screening assay after 48 hours of growth showing differences in fluorescence levels for clones with integrated eGFP, for each of the three target sites **A)** *04576*, **B)** *PFK1*, and **C)** *ROX1*. Data is shown as relative frequency histograms. All fluorescent clones with OD600>1 (n=892) were included in the analysis.

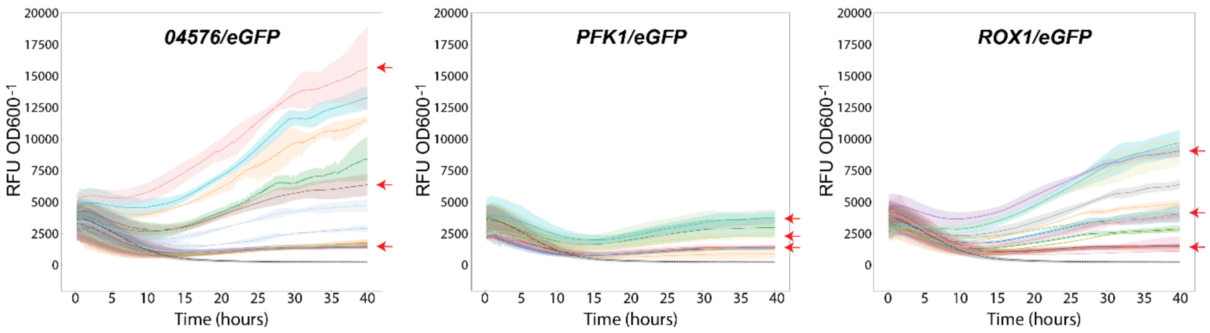

Supplementary Figure S3: Kinetic fluorescence growth assay for selected fluorescent clones. The data presented is the same as in Figure 4B, but standard deviations are shown as shading around the solid line indicating the averages of 2 to 4 biological replicates. Red arrows point at the nine isolates with different fluorescence levels (low, medium, strong; respectively, left panel: *04576*_35, *04576*_38, *04576*_37, centre panel: *PFK1*_72, *PFK1*_66, *PFK1*_21, right panel: *ROX1*_96, *ROX1*_87, *ROX1*_97) selected for WGS. Normalized fluorescence (RFU $OD600^{-1}$) is defined as relative fluorescence units (RFU) relative to culture optical density at 600 nm (OD600). The discontinuous black line corresponds to the wild-type control.

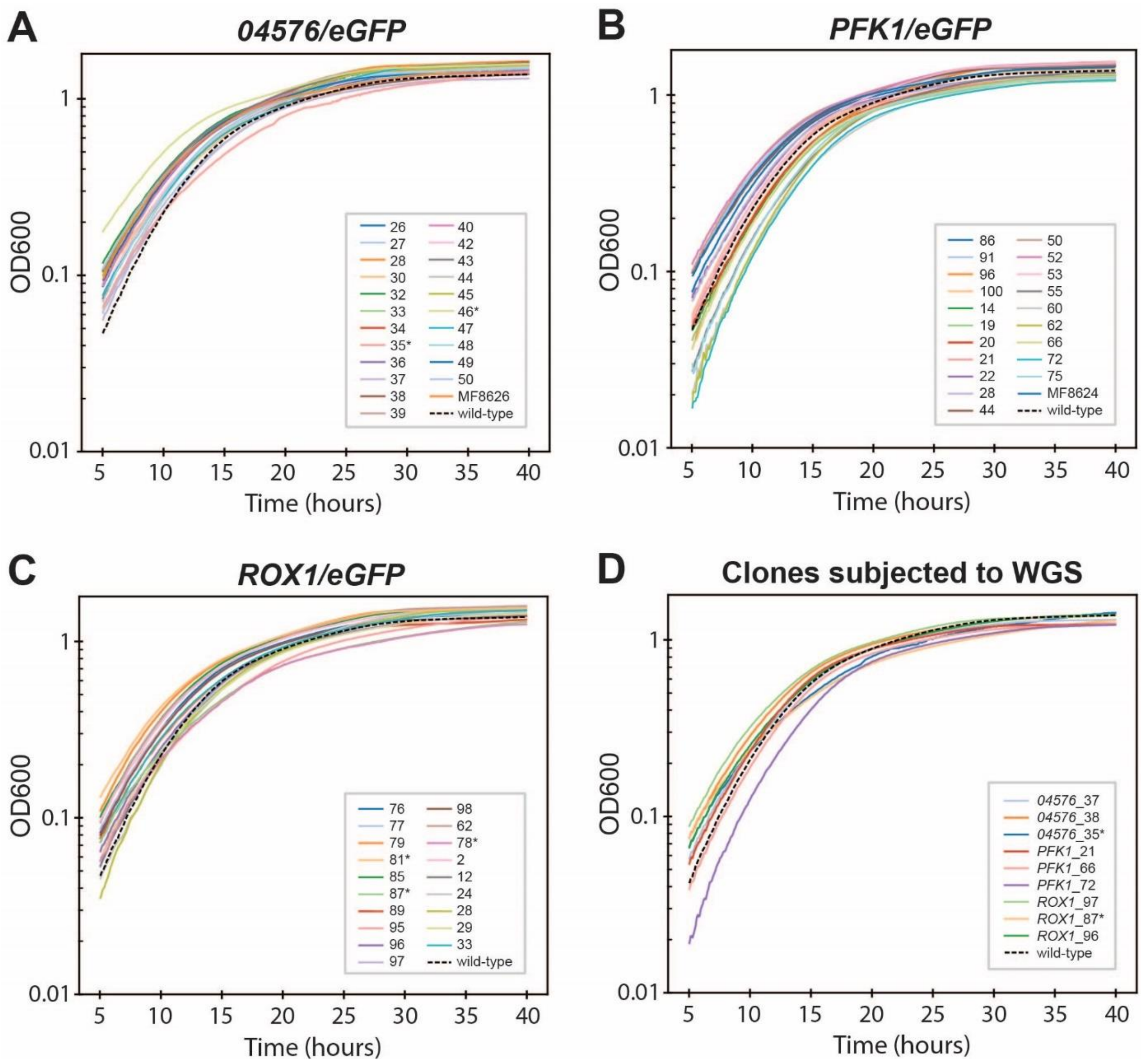

Supplementary Figure S4: Growth curves for clones subjected to the fluorescence growth assay. Each growth curve shows averages of 2 to 4 biological replicates. Relative fluorescence levels for the same clones are shown in Figure 4. Insertions were targeted into insertion sites **A)** *04576*, **B)** *PFK1*, and **C)** *ROX1*. **D)** Growth curves for the 9 clones subjected to WGS analysis. Legends indicate strain names. Strain names for the clones with a significantly lower growth rate compared with the wild-type control strain (see Supplementary Table S1) are labelled with an asterisk (*).

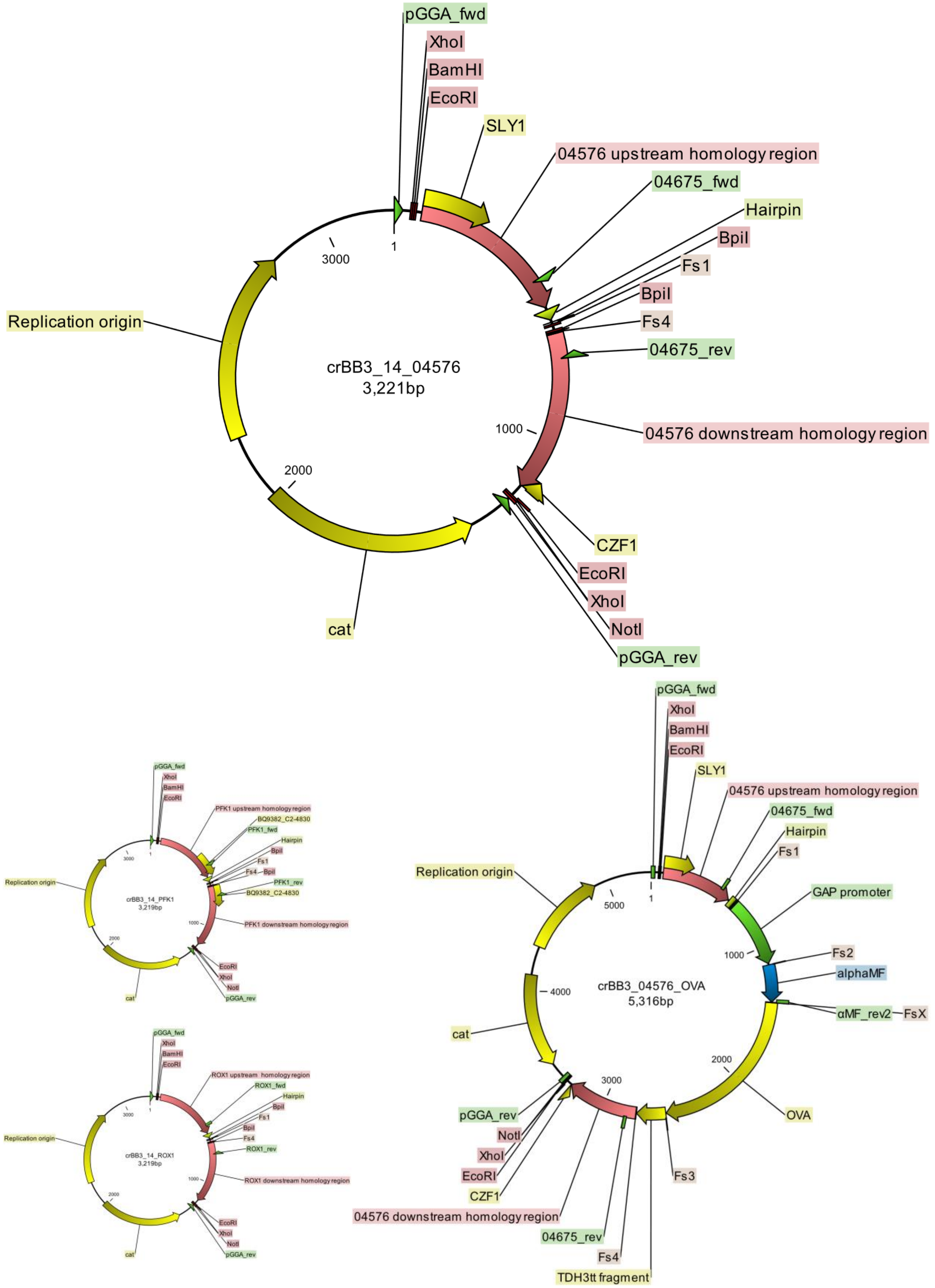

Supplementary Figure S5: Maps of key donor helper plasmids generated in this study. GenBank files for these plasmids are found in Supplementary File S2.

Supplementary Table S1: Summary of results from kinetic fluorescence growth assay with a wild-type control (WT; *Komagataella phaffii* CBS 7435) and approximately 20 randomly selected clones for each of three insertion sites (*04576*, *PFK1* and *ROX1*). Indicated in the table are average specific growth rates (μ; h^{-1}) calculated at exponential phase during periods representative for three different observed slopes (average±SE), as well as the maximum OD600 values reached at stationary phase (average±SE). Statistically significant differences after one-way ANOVA (normally distributed data, homoscedastic) or Kruskal-Wallis test (not normally distributed data) comparing growth rate to that of the control strain are indicated in brackets [p-value; statistically significant if p<0.05]. Clones presenting statistically significant growth rates are indicated in bold.

Clone ID	μ (h^{-1}) 5-7.5h after inoculation	μ (h^{-1}) 9-11h after inoculation	μ (h^{-1}) 11-13h after inoculation	Max OD600 at stationary phase
WT control	0.13 (SE±0.01)	0.17 (SE±0.00)	0.16 (SE±0.00)	1.57 (SE±0.05)
*04576*_26	0.16 (SE±0.01)	0.16 (SE±0.01)	0.13 (SE±0.01)	1.66 (SE±0.11)
*04576*_27	0.18 (SE±0.01)	0.14 (SE±0.01)	0.13 (SE±0.02)	1.81 (SE±0.08)
*04576*_28	0.17 (SE±0.02)	0.15 (SE±0.02)	0.13 (SE±0.02)	1.85 (SE±0.10)
*04576*_30	0.15 (SE±0.00)	0.15 (SE±0.01)	0.13 (SE±0.02)	1.75 (SE±0.04)
*04576*_32	0.16 (SE±0.00)	0.15 (SE±0.01)	0.13 (SE±0.02)	1.85 (SE±0.07)
*04576*_33	0.15 (SE±0.00)	0.15 (SE±0.01)	0.13 (SE±0.01)	1.80 (SE±0.09)
*04576*_34	0.15 (SE±0.01)	0.15 (SE±0.01)	0.13 (SE±0.01)	1.86 (SE±0.11)
***04576*_35**	0.13 (SE±0.01)	0.13 (SE±0.00) [p=0.0006]	0.12 (SE±0.01) [p=0.0036]	1.65 (SE±0.09)
*04576*_36	0.16 (SE±0.01)	0.15 (SE±0.01)	0.12 (SE±0.02)	1.75 (SE±0.05)
*04576*_37	0.13 (SE±0.01)	0.16 (SE±0.01)	0.15 (SE±0.01)	1.56 (SE±0.06)
*04576*_38	0.14 (SE±0.00)	0.15 (SE±0.00)	0.15 (SE±0.01)	1.59 (SE±0.06)
*04576*_39	0.16 (SE±0.01)	0.14 (SE±0.01)	0.13 (SE±0.02)	1.74 (SE±0.05)
*04576*_40	0.15 (SE±0.01)	0.16 (SE±0.02)	0.14 (SE±0.02)	1.56 (SE±0.14)
*04576*_42	0.15 (SE±0.01)	0.15 (SE±0.01)	0.14 (SE±0.01)	1.57 (SE±0.11)
*04576*_43	0.16 (SE±0.01)	0.15 (SE±0.00)	0.13 (SE±0.02)	1.69 (SE±0.05)
*04576*_44	0.15 (SE±0.00)	0.16 (SE±0.00)	0.15 (SE±0.01)	1.69 (SE±0.01)
*04576*_45	0.16 (SE±0.01)	0.15 (SE±0.02)	0.13 (SE±0.02)	1.71 (SE±0.02)
***04576*_46**	0.15 (SE±0.01)	0.13 (SE±0.01)	0.11 (SE±0.01) [p=0.0091]	1.78 (SE±0.10)
*04576*_47	0.14 (SE±0.00)	0.15 (SE±0.00)	0.14 (SE±0.00)	1.82 (SE±0.10)
*04576*_48	0.15 (SE±0.01)	0.16 (SE±0.01)	0.15 (SE±0.02)	1.80 (SE±0.08)
*04576*_49	0.15 (SE±0.01)	0.16 (SE±0.02)	0.14 (SE±0.02)	1.56 (SE±0.14)
*04576*_50	0.15 (SE±0.02)	0.15 (SE±0.02)	0.13 (SE±0.03)	1.55 (SE±0.16)
*04576*_MF 8626	0.17 (SE±0.01)	0.15 (SE±0.00)	0.12 (SE±0.00)	1.57 (SE±0.12)
*PFK1*_14	0.14 (SE±0.00)	0.16 (SE±0.00)	0.15 (SE±0.00)	1.47 (SE±0.07)
*PFK1*_19	0.13 (SE±0.01)	0.16 (SE±0.01)	0.15 (SE±0.00)	1.45 (SE±0.05)
*PFK1*_20	0.15 (SE±0.01)	0.15 (SE±0.01)	0.15 (SE±0.01)	1.55 (SE±0.15)
*PFK1*_21	0.14 (SE±0.01)	0.16 (SE±0.00)	0.15 (SE±0.01)	1.45 (SE±0.05)
*PFK1*_22	0.15 (SE±0.01)	0.16 (SE±0.01)	0.15 (SE±0.01)	1.52 (SE±0.11)
*PFK1*_28	0.15 (SE±0.01)	0.16 (SE±0.00)	0.14 (SE±0.00)	1.52 (SE±0.11)
*PFK1*_44	0.15 (SE±0.01)	0.15 (SE±0.01)	0.13 (SE±0.01)	1.66 (SE±0.04)
*PFK1*_50	0.16 (SE±0.01)	0.15 (SE±0.01)	0.12 (SE±0.00)	1.69 (SE±0.04)
*PFK1*_52	0.16 (SE±0.00)	0.15 (SE±0.01)	0.13 (SE±0.01)	1.72 (SE±0.02)

Clone ID	μ (h^{-1}) 5-7.5h post-inoculation	μ (h^{-1}) 9-11h post-inoculation	μ (h^{-1}) 11-13h post-inoculation	Max OD600 at stationary phase
*PFK1*_53	0.14 (SE±0.01)	0.17 (SE±0.01)	0.15 (SE±0.00)	1.70 (SE±0.04)
*PFK1*_55	0.11 (SE±0.03)	0.16 (SE±0.00)	0.16 (SE±0.01)	1.48 (SE±0.12)
*PFK1*_60	0.09 (SE±0.04)	0.14 (SE±0.02)	0.15 (SE±0.01)	1.39 (SE±0.04)
*PFK1*_62	0.10 (SE±0.02)	0.16 (SE±0.00)	0.17 (SE±0.00)	1.43 (SE±0.01)
*PFK1*_66	0.13 (SE±0.01)	0.16 (SE±0.00)	0.16 (SE±0.00)	1.43 (SE±0.02)
*PFK1*_72	0.12 (SE±0.00)	0.15 (SE±0.01)	0.16 (SE±0.00)	1.32 (SE±0.02)
*PFK1*_75	0.10 (SE±0.00)	0.16 (SE±0.00)	0.17 (SE±0.00)	1.38 (SE±0.01)
*PFK1*_86	0.15 (SE±0.01)	0.17 (SE±0.01)	0.14 (SE±0.01)	1.62 (SE±0.05)
*PFK1*_91	0.16 (SE±0.01)	0.15 (SE±0.00)	0.12 (SE±0.01)	1.69 (SE±0.00)
*PFK1*_96	0.14 (SE±0.01)	0.17 (SE±0.01)	0.15 (SE±0.01)	1.71 (SE±0.03)
*PFK1*_100	0.14 (SE±0.01)	0.17 (SE±0.01)	0.15 (SE±0.01)	1.54 (SE±0.09)
*PFK1*_MF8624	0.16 (SE±0.01)	0.16 (SE±0.01)	0.13 (SE±0.01)	1.60 (SE±0.02)
*ROX1*_12	0.13 (SE±0.01)	0.16 (SE±0.00)	0.14 (SE±0.01)	1.65 (SE±0.05)
*ROX1*_2	0.15 (SE±0.00)	0.16 (SE±0.03)	0.13 (SE±0.01)	1.67 (SE±0.03)
*ROX1*_24	0.12 (SE±0.01)	0.17 (SE±0.01)	0.15 (SE±0.00)	1.70 (SE±0.03)
*ROX1*_28	0.14 (SE±0.02)	0.17 (SE±0.01)	0.16 (SE±0.01)	1.68 (SE±0.02)
*ROX1*_29	0.14 (SE±0.01)	0.14 (SE±0.01)	0.13 (SE±0.01)	1.59 (SE±0.09)
*ROX1*_33	0.15 (SE±0.01)	0.15 (SE±0.00)	0.13 (SE±0.00)	1.64 (SE±0.09)
*ROX1*_62	0.17 (SE±0.00)	0.15 (SE±0.00)	0.12 (SE±0.00)	1.74 (SE±0.02)
*ROX1*_76	0.15 (SE±0.01)	0.16 (SE±0.01)	0.14 (SE±0.02)	1.57 (SE±0.16)
*ROX1*_77	0.14 (SE±0.01)	0.15 (SE±0.00)	0.14 (SE±0.01)	1.59 (SE±0.12)
***ROX1*_78**	0.14 (SE±0.00)	0.13 (SE±0.00) [p=000.5]	0.11 (SE±0.00) [p<0.00001]	1.42 (SE±0.06)
*ROX1*_79	0.18 (SE±0.01)	0.15 (SE±0.01)	0.12 (SE±0.01)	1.71 (SE±0.07)
***ROX1*_81**	0.16 (SE±0.01)	0.14 (SE±0.01)	0.11 (SE±0.00) [p=0.0383]	1.70 (SE±0.06)
*ROX1*_85	0.16 (SE±0.01)	0.15 (SE±0.01)	0.13 (SE±0.02)	1.68 (SE±0.06)
***ROX1*_87**	0.13 (SE±0.00)	0.12 (SE±0.00) [p<0.00001]	0.11 (SE±0.00) [p<0.00001]	1.39 (SE±0.05)
*ROX1*_89	0.14 (SE±0.01)	0.15 (SE±0.01)	0.14 (SE±0.01)	1.53 (SE±0.05)
*ROX1*_95	0.13 (SE±0.00)	0.13 (SE±0.00)	0.11 (SE±0.00)	1.55 (SE±0.00)
*ROX1*_96	0.14 (SE±0.00)	0.15 (SE±0.00)	0.14 (SE±0.01)	1.55 (SE±0.05)
*ROX1*_97	0.15 (SE±0.01)	0.15 (SE±0.01)	0.14 (SE±0.01)	1.54 (SE±0.06)
*ROX1*_98	0.15 (SE±0.01)	0.16 (SE±0.00)	0.14 (SE±0.01)	1.54 (SE±0.06)

Supplementary Table S2: Normalized and relative coverage of genetic elements of interest from read mapping coverage data obtained from mapping of WGS data for strong, medium, and low eGFP expression level clones from reactions targeting the ***04576*** insertion site against the **plasmid crBB3_04576_eGFP reference**.

	Position on reference		Mean normalized coverage			Coverage relative to *eGFP*		
**Name of gene or genetic region**	**from**	**to**	**strong**	**medium**	**low**	**strong**	**medium**	**low**
04576 upstream homology arm	21	520	29.5	9.5	1.9	20.6	6.6	1.3
GAP promoter	579	1055	20.3	6.5	2.5	14.1	4.5	1.7
eGFP gene	1065	1784	19.7	6.7	1.4	13.7	4.7	1.0
TDH3tt terminator fragment	1785	1990	22.9	8.4	2.9	16.0	5.9	2.0
04576 downstream homology arm	1995	2494	17.1	5.5	1.5	11.9	3.8	1.0
cat gene	2686	3345	16.7	4.4	0.0	11.6	3.1	0.0
Replication origin (ori)	3549	4137	16.4	4.7	0.0	11.4	3.3	0.0
Entire donor cassette	21	2494	21.6	7.1	1.9	15.1	5.0	1.3
cat / ori fragment (*Eco*RI fragment)	2530	4611	15.9	4.5	0.0	11.1	3.1	0.0

Supplementary Table S3: Normalized and relative coverage of genetic elements of interest from read mapping coverage data obtained from mapping of WGS data for strong, medium, and low eGFP expression level clones from reactions targeting the ***04576*** insertion site against the ***K. phaffii* CBS 7435 reference genome** modified to contain the eGFP expression cassette inserted into the *04576* target site.

	Position on reference		Mean normalized coverage			Coverage relative to *eGFP*		
**Name of gene or genetic region**	**from**	**to**	**strong**	**medium**	**low**	**strong**	**medium**	**low**
04576 upstream homology arm	1280758	1281257	19.9	7.9	1.5	13.9	5.5	1.0
GAP promoter	1280223	1280699	15.2	4.6	1.2	10.6	3.2	0.9
eGFP gene	1279494	1280213	19.7	6.7	1.4	13.7	4.7	1.0
TDH3tt terminator fragment	1279288	1279493	20.9	6.3	1.6	14.6	4.4	1.1
04576 downstream homology arm	1278784	1279283	17.1	5.4	1.4	11.9	3.8	1.0
Entire donor cassette	1278784	1281257	20.5	6.6	1.5	14.3	4.6	1.0

Supplementary Table S4: Normalized and relative coverage of genetic elements of interest from read mapping coverage data obtained from mapping of WGS data for strong, medium, and low eGFP expression level clones from reactions targeting the ***PFK1*** insertion site against the **plasmid crBB3_PFK1_eGFP reference**.

	Position on reference		Mean normalized coverage			Coverage relative to *eGFP*		
**Name of gene or genetic region**	**from**	**to**	**strong**	**medium**	**low**	**strong**	**medium**	**low**
PFK1 upstream homology arm	21	518	4.8	3.1	1.5	3.4	2.2	1.1
GAP promoter	577	1053	5.4	4.2	2.3	3.8	2.9	1.6
eGFP gene	1063	1782	4.6	3.0	1.4	3.3	2.1	1.0
TDH3tt terminator fragment	1783	1988	7.2	4.7	3.4	5.1	3.3	2.4
PFK1 downstream homology arm	1993	2492	6.7	3.8	2.1	4.7	2.7	1.5
cat gene	2684	3343	3.7	0.0	0.0	2.6	0.0	0.0
Replication origin (ori)	3547	4135	3.9	0.0	0.0	2.7	0.0	0.0
Entire donor cassette	21	2492	5.4	3.5	1.9	3.8	2.5	1.3
cat / ori fragment (*Eco*RI fragment)	2528	4609	3.7	0.0	0.0	2.6	0.0	0.0

Supplementary Table S5: Normalized and relative coverage of genetic elements of interest from read mapping coverage data obtained from mapping of WGS data for strong, medium, and low eGFP expression level clones from reactions targeting the ***PFK1*** insertion site against the ***K. phaffii* CBS 7435 reference genome** modified to contain the eGFP expression cassette inserted into the *PFK1* target site.

	Position on reference		Mean normalized coverage			Coverage relative to *eGFP*		
**Name of gene or genetic region**	**from**	**to**	**strong**	**medium**	**low**	**strong**	**medium**	**low**
PFK1 upstream homology arm	1653952	1654449	4.8	3.1	1.6	3.4	2.2	1.1
GAP promoter	1653417	1653893	3.2	2.3	1.2	2.3	1.6	0.8
eGFP gene	1652688	1653407	4.6	3.0	1.4	3.3	2.1	1.0
TDH3tt terminator fragment	1652482	1652687	5.7	3.1	1.9	4.0	2.2	1.4
PFK1 downstream homology arm	1651978	1652477	6.7	3.9	2.1	4.8	2.8	1.5
Entire donor cassette	1651978	1654449	4.9	3.1	1.6	3.5	2.2	1.1

Supplementary Table S6: Normalized and relative coverage of genetic elements of interest from read mapping coverage data obtained from mapping of WGS data for strong, medium, and low eGFP expression level clones from reactions targeting the ***ROX1*** insertion site against the **plasmid crBB3_ROX1_eGFP reference**.

	Position on reference		Mean normalized coverage			Coverage relative to *eGFP*		
**Name of gene or genetic region**	**from**	**to**	**strong**	**medium**	**low**	**strong**	**medium**	**low**
ROX1 upstream homology arm	21	520	12.5	3.4	1.7	8.6	2.3	1.2
GAP promoter	579	1055	10.5	3.7	2.6	7.2	2.6	1.8
eGFP gene	1065	1784	11.5	2.7	1.5	7.9	1.9	1.0
TDH3tt terminator fragment	1785	1990	14.1	5.7	3.4	9.7	3.9	2.3
ROX1 downstream homology arm	1995	2492	15.6	4.1	1.9	10.8	2.8	1.3
cat gene	2684	3343	10.2	7.5	0.0	7.0	5.1	0.0
Replication origin (ori)	3547	4135	9.5	7.1	0.0	6.6	4.9	0.0
Entire donor cassette	21	2492	12.5	3.6	2.0	8.6	2.5	1.4
cat / ori fragment (*Eco*RI fragment)	2528	4609	10.0	7.0	0.0	6.9	4.8	0.0

Supplementary Table S7: Normalized and relative coverage of genetic elements of interest from read mapping coverage data obtained from mapping of WGS data for strong, medium, and low eGFP expression level clones from reactions targeting the ***ROX1*** insertion site against the ***K. phaffii* CBS 7435 reference genome** modified to contain the eGFP expression cassette inserted into the *ROX1* target site.

	Position on reference		Mean normalized coverage			Coverage relative to *eGFP*		
**Name of gene or genetic region**	**from**	**to**	**strong**	**medium**	**low**	**strong**	**medium**	**low**
ROX1 upstream homology arm	2361317	2361816	12.6	3.5	1.7	8.6	2.4	1.2
GAP promoter	2360782	2361258	7.4	2.4	1.3	5.1	1.7	0.9
eGFP gene	2360053	2360772	11.5	2.7	1.5	7.9	1.9	1.0
TDH3tt terminator fragment	2359847	2360052	12.6	4.0	1.9	8.7	2.7	1.3
ROX1 downstream homology arm	2359345	2359842	15.6	4.1	2.0	10.7	2.8	1.3
Entire donor cassette	2359345	2361816	11.8	3.2	1.6	8.1	2.2	1.1

Supplementary Table S8: PCR primers for verification of insertions

Primer name	Sequence (5' to 3')	Target sequence and application
pGAP_fwd	GTCTCTTCCCTTCTCTCTCC	Flanking the sgRNA insertion site on the in the CRIS*Pi* plasmids.
RPS25_rev	CCGGGTAACTTTAGGAGGAT	
pGGA_fwd	CTGCAGGAAGGTTTAAACGCATTTAGG	Flanking the insertion site for homology regions on the pGGAselect plasmid. Used during crBB3 donor helper plasmid construction.
pGGA_rev	TAATACGACTCACTATAGGGAGACTC	
04675_fwd	AGCTTGCAGATTGCTGAATG	Flanking the expression cassette insertion sites in crBB3_14_04576.
04675_rev	GATTGCTGACCGCTTCTGAG	
PFK1_fwd	CCCTCAATTGGTTGCATACTG	Flanking the expression cassette insertion sites in crBB3_14_PFK1.
PFK1_rev	CGGTATGATCGTTTGCATTG	
ROX1_fwd	CTTGTAGCGGGCAGGACTT	Flanking the expression cassette insertion sites in crBB3_14_ROX1.
ROX1_rev	CCAGCACGGATCATAGGAGA	
out_04576_fwd	ACCAAGTCTGGCCACTCAAG	Outside of the *04576* homology regions on the *K. phaffii* genome.
out_04576_rev	GCCAGCTCCTGGTACTGAAA	
out_PFK1_fwd	CCTCTTGAGTGGTCGTTTGTC	Outside of the *PFK1* homology regions on the *K. phaffii* genome.
out_PFK1_rev	CTGTGAAATGTCAGCTGGTTT	
out_ROX1_fwd	GAGTGAGGCTCATTTCAGTGG	Outside of the *ROX1* homology regions on the *K. phaffii* genome.
out_ROX1_rev	AGCCTGGAGGTTTAGATGGA	
aMF_rev	AGCTTCAGCCTCTCTTTTCTCT	Binds to α-MF within the donor DNA cassette. Used with out_04576_fwd, out_PFK1_fwd, or out_ROX1_fwd to cover the 5’ side of the integration locus.

Supplementary Table S9: Single guide RNAs used in this study

	Target gene	Sequence
sgRNA-G	Near *GQ67-04576*	CTGTGCGTGCCGTGACACCT
sgRNA-H	Near *PFK1*	ACTCTTTTCGCTGTGATGAT
sgRNA-I	Near *ROX1*	CACGTAGCCCCGGCGTCCTC

Reference: Dalvie et al. 2020, *ACS Synth Biol*, 9(1), 26-35.

Supplementary Table S10: Oligonucleotide sequences for construction of the HH-sgRNA-HDV fusion genes

Oligo/primer name	Sequence (5' to 3')
sgRNA_fw_04576	t**gaagac**gccatg**GCACAG**CTGATGAGTCCGTGAGGACGAAACGAGTAAGCTCG TC**CTGTGCGTGCCGTGACACCT**GTTTTAGAGCTAGAAATAGCAAG
sgRNA_fw_PFK1	t**gaagac**gccatg**AAGAGT**CTGATGAGTCCGTGAGGACGAAACGAGTAAGCTCG TC**ACTCTTTTCGCTGTGATGAT**GTTTTAGAGCTAGAAATAGCAAG
sgRNA_fw_ROX1	t**gaagac**gccatg**TACGTG**CTGATGAGTCCGTGAGGACGAAACGAGTAAGCTCG TC**CACGTAGCCCCGGCGTCCTC**GTTTTAGAGCTAGAAATAGCAAG
sgRNA_struc_rev	a**gaagac**gcaagCAGTCCAAAGCTGTCCCATTCGCCATGCCGAAGCATGTTGCC CAGCCGGCGCCAGCGAGGAGGCTGGGACCATGCCGGCCAAAAGCACCGACTCGG TGCCACTTTTTCAAGTTGATAACGGACTAGCCTTATTTTAACTTGCTATTTCTA GCTCTAAAAC
sgRNA_fw_04576_short	t**gaagac**gccatg**GCACA**
sgRNA_fw_PFK1_short	t**gaagac**gccatg**AAGAGT**CT
sgRNA_fw_ROX1_short	t**gaagac**gccatg**TACGTG**C
sgRNA_struc_rev_short	a**gaagac**gcaagCAGTCCAA

The *Bpi*I (*Bbs*I) recognition sites (gaagac) are shown in bold red. The fusion sites are underlined and the structural sequence (HV-sgRNA-HDV) is in uppercase letters. The target sequence is shown in blue bold. The 6 nucleotides following the forward primer fusion site, complementary to the start of the target sequence and essential for hammerhead processing, are shown in green bold.

Supplementary Table S11: Nucleotide sequences used for construction of the crBB3 plasmids

Fragment	Sequence (5' to 3')
04576_up	gatc**ggtctc**cggagagcaatacaacaattctattgtgtttgttgtaggagggggcaactatttggagtaccaa<br>aatttgcaagaatgggttaccaagaccaatactagcaacgtcaatggcactaagtctgtaatctacggtagtac<br>cagtatcgtgaccgcgaacgagttcttgaaggagtgctccttgctcggtgccgaagcaaaataaaagtttggaa<br>tcatatcatagtgcttatctcggaattttccgtcctgtgagatcttgtgttaccaatactgaacacagaggggа<br>ggaagagatttgagctgtttttcttttttgatgacgtatgctaagggcctatctgtcactgtaaagtttgg<br>atagcccagaagtctgtttgatacacatatgcgaggtgtcctgttattactcatacttagcttgcagattgctg<br>aatgtgtatagcccatgattcacagtaagcctggaagtcgacgttatcgacgtgctgtgcgtgccgtgacagct<br>tc**gagacc**gatc
04576_down_fw	tgac**ggtctc**gcgcttttggtcccgttttgagg
04576_down_rev	ggtc**ggtctc**catggaaaaattcttagaggcattgttg
PFK1_up	gatc**ggtctc**cggagtctttctctcgagctggatgaaactagtgcatgtacgaatccgtgtgtaatctactggg<br>atgcttattttacgcattttgttgataaaaatagagtcctactactactcctgatttcaagcctttctacctg<br>taagtttcttttttttttgctggtgacaatagcctttttttttacctttttgccatcgttgcgtcctgtatag<br>cttcttaaatgtctccgcaacaattttatgggagcaatagattacctctggttttactcgtaacctctagggt<br>taataggcccattaaattggctatgggaatgcgagtgatatggcttcaactatagagagtgcggattattgttc<br>atttctactgtatgcaagatgtggcatttttcttattgtggtgaaaggtaccgctgcccctcaattggttgca<br>tactgaaacgacaagttgagtcgagtccctggaaaatcctaccagttgcaggcaaaataagtcaaccacgcttc<br>**gagacc**gatc
PFK1_down	gatc**ggtctc**gcgctatcacagcgaaaagagttgcatttggttttgagagacggtgttttggtaaacagcaagc<br>cccaatgcaaacgatcataccgatctgccatggcagcctgttggtcacatgaaacacacaggagcacaggggaa<br>gttttattatcatgcgcggtgcaataaatgtgcatgagttacgcatttcggatatctgctccttatataaaagt<br>gtttacagataccatagtatgatgcgcgtatagtctctgtatctaccttgcttttgttctgcacaacaccacat<br>ttttcttggaccacttctagcataatccgtctgtgaaaaatgcacaccgcacttatctttcacgtatttgaaga<br>aaagccttttcatcgatcgaatgatattgcacttggacgcacgaacacacccattaccaataagaatgttcaa<br>aaaaaaacaaccatcatgcacagatcacattgcacctgataacttgtggcattatttctgcttctttaacccca<br>tc**gagacc**gatc
ROX1_up	gatc**ggtctc**cggagttgtgtttactcgattgtcgtgggaaagactttttcttttgatagtagagcaaaggaat<br>gcgcaccctcgcgcaagtggcattcacataatgcagttactctcgcatgttgttttgtaatcgaacgaaagtgc<br>caatgcagttccttcaatctagccaatttaccgattgttagagcagcaaaagagtggtactattgctctccgac<br>ttgaaattgaccactacaatatgtcatcttaacgcattggttactggatcgctctcgactcgtaatgtttaatt<br>ccgtttgaattaacctgcattcaacaaaaatgccttctgaatgaatgaccagtcctcaacatgtaattcatgcc<br>gccgagacttccaaaggaggcaaattccaaatagaccaaatgatcgccgccattcttgaataatcttagctaag<br>ccttgtagcgggcaggactttctgacctaagttactaaatcaaatgtatggcgtcacgtagccccggcgtcgct<br>tc**gagacc**gatc
ROX1_down	gatc**ggtctc**gcgcttcgcaggacccaaagccttcaaacaagcttaagtttctttgaagaacttttgtttagaa<br>aggtatctcatctattctcctatgatccgtgctgggcagtcaatcttcttgtatgccaggttgtcatctaaaaa<br>taaacaggtatcccactccagtagacacttgatctggttcaaattcagaaaaggtgacagcagacattgttgat<br>ttcttcagaatactctccagtatagcctctgcaagaagagatgaatctctggatgcaatagttagctgtagagc<br>cttgtgtttgaacaaccttgaggccatctgtgttgagcaaggctgataaaattcaggaacatacccaaagcttg<br>gcactgtcacgtaggtccactatttttgaccgctaccaagtctccttgataaagcccttcgttcatggtccttt<br>gaggccttagtgactcgtttgaacttgagttcctatccttactggagtgaaaaaaagccaagtgtccagccatc<br>**gagacc**gatc
Fs1-Fs4 linker	gatc**ggtctc**cgcttcgc**aaaaaaccccgcccctgacagggcggggtttttt**cgcgatcggaggc**gtcttc**gga<br>tccctcgag**gaagac**gccgctg**gagacc**gatc
FsA-FsC linker	gatc**gaagac**gcggaggatcg**gagacc**ggatccctcgag**ggtctc**caattcgctgc**gtcttc**gatc

The *Bpi*I (*Bbs*I) recognition sites (gaagac) are shown in bold red, *Bsa*I recognition sites (ggtctc) are shown in bold pink. The fusion sites are underlined. The artificial transcriptional terminator in the linker is in blue bold. Bases changed (relative to original sequence) to remove *Bpi*I, *Bsa*I, and *Eco*RI restriction sites are highlighted in green. The poly(dA·dT) sequence in the *PFK1* upstream region is highlighted in yellow.

Supplementary Table S12: Nucleotide sequences used for construction of the *OVA* gene expression cassette plasmid

Fragment	Sequence (5' to 3')
α-MF	gatc**gaagac**gctatgagatttccttcaatttttactgcagttttattcgcagcatcc<br>tccgcattagctgctccagtcaacactacaacagaagatgaaacggcacaaattccgg<br>ctgaagctgtcatcggttactcagatttagaaggggatttcgatgttgctgttttgcc<br>attttccaacagcacaaataacgggttattgtttataaatactactattgccagcatt<br>gctgctaaagaagaaggggtatctttagagaaaagagaggctgaagctgc**gtcttc**ga<br>tc
aMF-fwd-Fs2	atc**gaagac**gccatgagatt
aMF-rev-FsD	att**gaagac**gcagcttcag
OVA	gatc**gaagac**gcagctggttctattggagcagcatcaatggagttttgctttgacgtg<br>tttaaggaacttaaggtgcatcatgccaatgagaacattttctactgtccaatagcca<br>ttatgtctgccttagctatggtctatttaggagctaaggatagtaccagaactcagat<br>taacaaggtcgttcgttttgacaaattaccaggatttggcgattctatagaggcccaa<br>tgtggtacgagtgttaacgtgcactcaagccttagggacattctaaaccagattacca<br>aaccgaatgacgtgtactcgttctccttggcttccagattatatgccgaggaaagata<br>cccaatacttcctgaatatctgcaatgtgttaaggagttgtatcgaggtggtcttgaa<br>cctatcaacttccagactgctgctgatcaagctagagaactgattaattcctgggttg<br>aatcccaaactaacggtattatccgaaacgttttgcaaccatcttcagtcgatagtca<br>aactgcaatggtcctagttaatgctatcgttttcaaaggtctgtgggagaaagccttt<br>aaggatgaagatacacaagctatgccctttagagttaccgaacaagagtccaaaccag<br>tacagatgatgtaccagattggcttgttcagagttgcttcaatggcaagcgagaaaat<br>gaagatcttggagttgccttttgcaagtggcacaatgtccatgcttgtgttgttgcca<br>gatgaagtttctggattagaacagttggagagcatcatcaactttgagaaactgactg<br>aatggacatcctcaaacgtaatggaagagaggaaaattaaggtctatctacctagaat<br>gaaaatggaagagaagtacaatttgacctcagtcttgatggctatggggattactgac<br>gtgttctcttcgtctgccaatttgagtgggatttcttccgctgaaagtctgaagattt<br>ctcaagctgtacatgcagcacatgccgagatcaatgaagctggtagagaagttgtagg<br>atcagctgaagctggtgttgacgctgcttctgtctctgaagaatttagagcagatcac<br>cctttcctattctgcataaagcacatcgccacgaatgctgttttgttctttggacgtt<br>gtgtatcgccctaagcttgc**gtcttc**gatc

The *Bpi*I (*Bbs*I) recognition sites (gaagac) are shown in bold red. The fusion sites are underlined. Bases changed (relative to original sequence) to remove the XhoI restriction site or change the fusion site is in green highlighting.

Supplementary Table S13: Analysis of Variance for transformation efficiency

Source	DF	Adj SS	Adj MS	F-Value	P-Value
Strain (3 levels: *04576*, *PFK1*, and *ROX1*)	2	12072033	6036017	19.16	0.000
Timepoint (3 levels: 1h, 2h, and 3h)	2	3074903	1537452	4.88	0.019
Experiment (3 levels: Replicates 1, 2, and 3)	2	4862824	2431412	7.72	0.003
Error	20	6301174	315059		
Total	26	26310934			

Supplementary Table S14: Tukey Simultaneous Tests for Differences of Means for transformation efficiency

Difference of strain Levels	Difference of Means	SE of Difference	Simultaneous 95% CI	T-Value	Adjusted P-Value
PFK1 - 04576	819	265	(149; 1489)	3.10	0.015
ROX1 - 04576	1638	265	(968; 2308)	6.19	0.000
ROX1 - PFK1	819	265	(149; 1489)	3.10	0.015

Individual confidence level = 98,01%

Supplementary Table S15: Analysis of Variance for integration efficiency

Source	DF	Adj SS	Adj MS	F-Value	P-Value
Strain (3 levels: *04576*, *PFK1*, and *ROX1*)	2	0.75713	0.378565	87.81	0.000
Timepoint (3 levels: 1h, 2h, and 3h)	2	0.02395	0.011977	2.78	0.086
Experiment (3 levels: Replicates 1, 2, and 3)	2	0.06418	0.032090	7.44	0.004
Error	20	0.08622	0.004311		
Total	26	0.93148			

Supplementary Table S16: Tukey Simultaneous Tests for Differences of Means for integration efficiency

Difference of strain Levels	Difference of Means	SE of Difference	Simultaneous 95% CI	T-Value	Adjusted P-Value
PFK1 - 04576	-0.4093	0.0310	(-0.4877; -0.3310)	-13.22	0.000
ROX1 - 04576	-0.2276	0.0310	(-0.3060; -0.1493)	-7.35	0.000
ROX1 - PFK1	0.1817	0.0310	(0.1034; 0.2601)	5.87	0.000

Individual confidence level = 98,01%

Supplementary Table S17: Analysis of Variance for specific growth rates in the kinetic fluorescence growth assay calculated during the **first** exponential period (5-7.5 h after inoculation)

Source	DF	SS	MS	F (DFn, DFd)	P-Value
Growth rate	62	0.04604	0.0007426	F (62,150) = 2.372	<0.0001
Error	150	0.04696	0.0003131		
Total	212	0.09301			

Supplementary Table S18: Analysis of Variance for specific growth rates in the kinetic fluorescence growth assay calculated during the **second** exponential period (9-11 h after inoculation)

Source	DF	SS	MS	F (DFn, DFd)	P-Value
Growth rate	62	0.02766	0.0004461	F (62,155) = 2.406	<0.0001
Error	155	0.02873	0.0001854		
Total	217	0.05639			

Supplementary Table S19: Analysis of Variance for specific growth rates in the kinetic fluorescence growth assay calculated during the **third** exponential period (11-13 h after inoculation)

Source	DF	SS	MS	F (DFn, DFd)	P-Value
Growth rate	62	0.04781	0.0007711	F (62,155) = 2.545	<0.0001
Error	155	0.04696	0.0003030		
Total	217	0.09477			
